# Two-Chamber Aminocarbonylation of Aryl Bromides and Triflates Using Amino Acids as Nucleophiles

**DOI:** 10.1021/acs.joc.3c00972

**Published:** 2023-08-28

**Authors:** Jens Lindman, Anubha Yadav, Johan Gising, Mats Larhed

**Affiliations:** Department of Medicinal Chemistry, Uppsala University, Husargatan 3, SE-751 23 Uppsala, Sweden

## Abstract

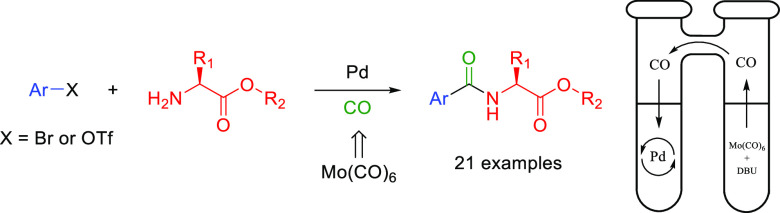

A palladium(0)-catalyzed aminocarbonylation reaction employing molybdenum hexacarbonyl as a carbon monoxide precursor for the production of N-capped amino acids using aryl and heteroaryl bromides and triflates is reported. The carbon monoxide is formed *ex situ* through the use of a two-chamber system, where carbon monoxide generated in one chamber is free to diffuse over and be consumed in the other palladium-catalyzed reaction chamber. Using this method, two series of aryl bromides and aryl triflates were utilized to synthesize 21 N-capped amino acids in isolated yields between 40 and 91%.

## Introduction

Peptide drugs have in the past decades emerged as an important tool in the treatment of a vast amount of diseases, such as cancer^1^ and diabetes^2^ in addition to their use as diagnostic agents.^3,4^ Peptide-containing drugs today constitute a large part of newly registered drugs.^5^ They allow the possibility for high potency, biological specificity, low toxicity, and a relatively low cost of manufacture. However, the use of peptides as drugs has long been riddled with problems in regards to low bioavailability, often forcing parenteral administration, as well as related low metabolic stability. Several ways to overcome these issues through chemical modifications of the peptides have been devised, such as PEGylation,^6^ N- and C-terminal functionalization,^7−9^ cyclization,^10^ and D -amino acid insertion.^11,12^*N*-aroylation- or heteroaroylation of amino acid residues is a strategy that has been used in a number of medicinal chemistry projects aimed at the development of bioactive peptides, where this strategy can improve affinity, reduce metabolic degradation as well as capping of basic amino groups.^8,9,13,14^

In this work, we employ a palladium(0)-catalyzed aminocarbonylation,^15,16^ in order to obtain *N*-aroylated- or heteroaroylated amino acids. The usual way of *N*-aryolating amino acids and peptides involves the activation of an aromatic carboxylic acid through the use of peptide coupling reagents or other means of carboxylic acid activation, such as the formation of acid chlorides, which are subsequently attacked by the amino acid amine. Our method provides an alternate route for the synthesis of *N*-aroylated amino acids that removes the need of carboxylic acid activation. Although the use of carbon monoxide gas has previously been used for palladium-catalyzed aminocarbonylation of aryl bromides employing amino acids,^17,18^ the hazards associated with the use of pressurized cylinders with highly toxic and flammable carbon monoxide have prompted the development of a variety of precursors, which release carbon monoxide in situ in a controlled fashion followed by simultaneous consumption.^19−27^ Earlier studies describing aminocarbonylation using such carbon monoxide precursors for N-capping of amino acids have primarily employed aryl iodides, which while requiring milder conditions due to the relatively facile oxidative addition, are less common and more expensive than the aryl bromides and readily available triflates used in this work.^28,29^ We decided to use molybdenum hexacarbonyl, a solid compound that is well-known to release carbon monoxide by decomposition at high temperatures or by ligand exchange in the presence of a strong base such as 1,8-diazabicyclo(5.4.0)undec-7-ene (DBU).^30^ As molybdenum hexacarbonyl is not inert and by itself can catalyze reactions such as the reduction of nitro groups,^31^ we utilize a two-chamber system for carbonylation.^23^ The ex situ carbon monoxide production and the palladium-catalyzed carbonylation are separated into different vials that are fused together with a bridge, allowing for the diffusion of carbon monoxide between the vials.^23,32^

Using this method, we synthesize 21 *N*-aroyl or -heteroaroylated amino acids from aryl and heteroaryl bromides and triflates, which to our knowledge have not been used as precursors in previous aminocarbonylation reactions with amino acids.

## Results and Discussion

The study was initiated by conducting a reaction with bromobenzene (**1a**, 0.64 mmol, 1 equiv) and l-phenylalanine *tert*-butyl ester hydrochloride (**2a**) with 5 mol % Pd(OAc)_2_, 10 mol % XantPhos and triethylamine (3 equiv) in the first chamber of the H-tube and molybdenum hexacarbonyl ((Mo(CO)_6_), 2 equiv) and DBU (2 equiv) in the other chamber with DMF (2 mL per chamber) used as solvent with heating at 100 °C for 2 h.^33^ The results of this initial experiment, which resulted in a 75% isolated yield of **3a** (Table 1, entry 1), prompted us to continue screening the reaction conditions, beginning with altering the palladium ligand. From the tested ligands (entries 2–5), none performed as well as XantPhos. The bidentate ligands dppf and dppe did not give any isolated yield of the product and liquid chromatography-ultraviolet/mass spectrometry (LC-UV/MS) analysis of the crude reaction mixtures revealed significant amounts of remaining starting material. DpePhos, which is similar to XantPhos but with a smaller bite angle owing to the lack of the xanthene bridge carbon, lead to a significant reduction of product yield. Attempts to increase or decrease the amount of amino acid loading in the reaction mixture did not lead to any large differences in the amount of product formed (entries 6 and 7), however, due to this finding, we could continue our screening with a reduced amino acid to bromobenzene ratio. Next, we looked at the Pd(OAc)_2_ loading (entries 8 and 9), where reducing the loading from 5 to 1 mol % provided a marginal increase in yield from 75 to 82%. Going in the opposite direction by increasing the loading to 10 mol % did not have any effect on the reaction outcome. As we planned to include other esters than *tert*-butyl esters in our substrate scope, we replaced the amino acid in the reaction screening with l-leucine methyl ester hydrochloride (**2d**) and tried it with the best-performing conditions and found it to perform similarly to the previously used **2a** (entry 10). We then evaluated further reduction of the Pd(OAc)_2_ loading to 0.1 mol %, which was found to drastically reduce the conversion (entry 11). Next, we investigated the effect of changing reaction time (entries 12 and 13) by running a reaction with the best conditions achieved so far for 30 min, providing 33% isolated yield after isolation by flash chromatography. Instead increasing the reaction time to 4 h furnished a significantly improved outcome, with an isolated yield of 88%. In an attempt to find a replacement for dimethylformamide (DMF), a series of solvents was investigated (entries 14–16). The reaction was well accommodated in the green solvent 2-methyltetrahydrofuran^34^ (entry 14), with an isolated yield after 4 h of 80%. Unfortunately, the reaction was hampered by the formation of palladium black, a concerning sign of an unstable catalytic system, which had not been encountered in the previous reactions in DMF. The reaction also proceeded in acetonitrile but with much lower conversion to product (entry 15). Trace amounts of the product could be observed upon LC-UV/MS analysis of the reaction run in 1,4-dioxane, but none was isolated through chromatography (entry 16). In addition, in the reaction using 1,4-dioxane the formation of palladium black was evident, indicating a loss of the active catalytic species. An attempt at conducting the reaction using a conventional one-chamber system, where the Mo(CO)_6_ and DBU were mixed with the other reaction components in a sealed reaction vial resulted in only trace amounts of product **3a** as monitored by LC-UV/MS (entry 17).

**Table 1:**
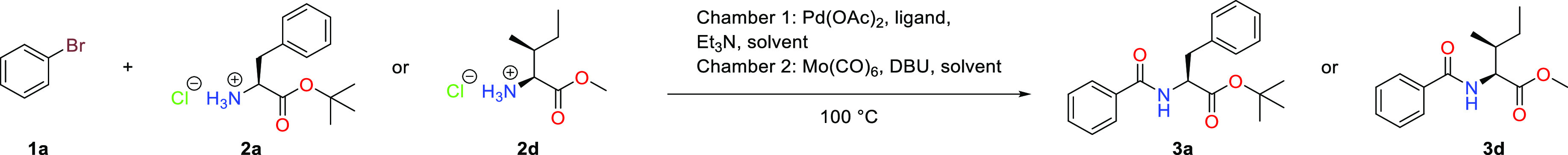

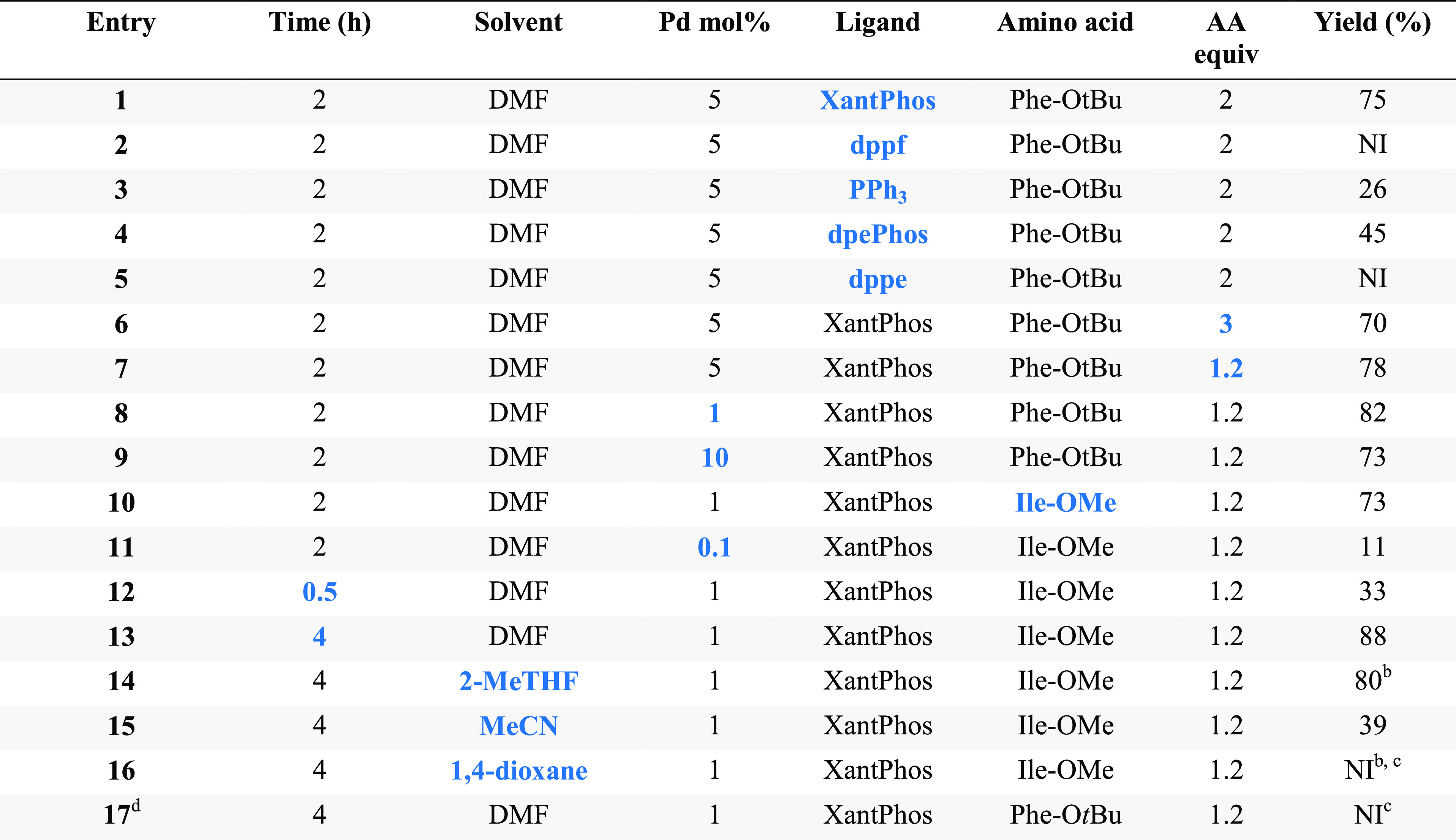
Optimization of Reaction Conditions for the Palladium(0)-Catalyzed Aminocarbonylation Reaction^a^

a Reaction conditions: Fused 5 mL vials. Chamber 1: Bromobenzene (0.64 mmol, 1 equiv), phenylalanine *tert*-butyl ester hydrochloride or isoleucine methyl ester hydrochloride, Pd(OAc)_2_, ligand (2 times the mol % of palladium), triethylamine (3 equiv), and solvent (2 mL). Chamber 2: Mo(CO)_6_ (2 equiv), DBU (3 equiv), and solvent (2 mL).

b Large amounts of palladium black formation.

c Trace amounts of product **3a** or **3d** detected through LC–MS analysis.

d Reaction conducted in a one-chamber system. NI = no product isolated. The parameter changed between experiments is marked in blue.

**Scheme 1:**
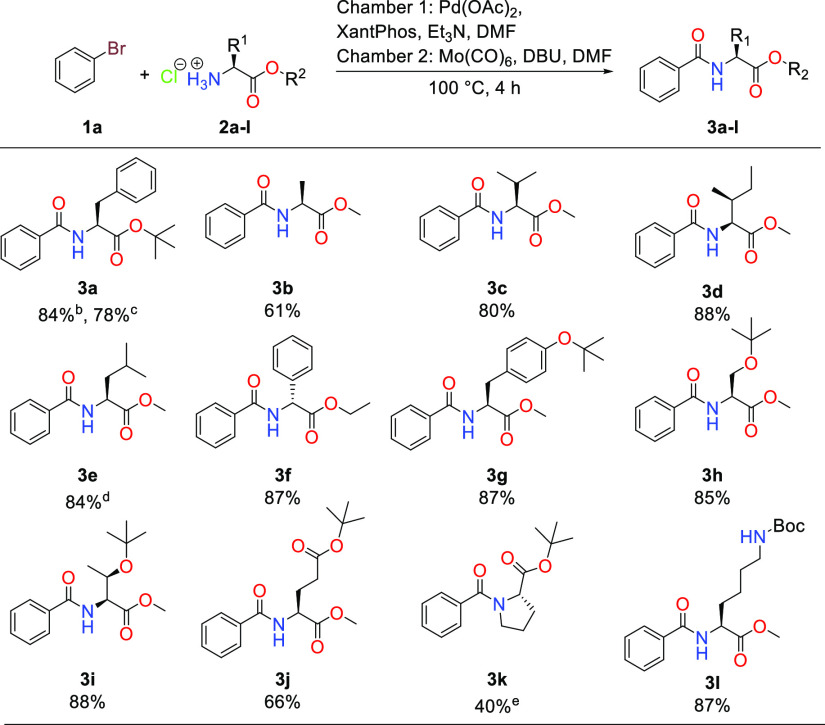
Substrate Scope with Regards to Amino Acid Ester Reaction conditions for **3a**–**3n**: Chamber 1: Bromobenzene (1 equiv), amino acid ester hydrochloride (1.2 equiv), Pd(OAc)_2_ (1 mol %), XantPhos (2 mol %), triethylamine (3 equiv), and DMF. Chamber 2: Mo(CO)_6_ (2 equiv), DBU (3 equiv), and DMF. Isolated yields. No racemization detected. Reaction conducted on a 1.5 mmol scale. 90% ee. With the addition of a catalytic amount of DMAP.

Applying the optimal conditions from the reaction screening, we investigated the scope with respect to the amino acid (Scheme 1). All amino acids bearing nonpolar side chains with the exception of alanine methyl ester **3b** (61%) were isolated in yields exceeding 80% (entries **3b**–**e**). The nonproteinogenic d-phenylglycine performed similarly and was isolated in 87% yield of **3f**. A synthesis of **3a** was also conducted on a 1.5 mmol scale, resulting in a yield of 78%. Trials with tyrosine methyl ester with the hydroxyl group unprotected gave rise to a mixture of products, presumably due to the phenol being deprotonated under the basic conditions, competing with the primary amine and giving rise to ester formation after nucleophilic attack on the acyl palladium intermediate (results not shown). Protection of the phenol as a *tert*-butoxy group remedied this issue, and the desired product could be isolated in 87% yield (**3g**). This *tert*-butyl side-chain protection strategy was also successfully used for aliphatic alcohol- and carboxyl-containing amino acids **3h**–**j**. Products derived from serine and threonine were produced in good yields (**3h** and **3i**, 85 and 88%, respectively), but the yield decreased slightly to 66% for the corresponding glutamic acid product (**3j**). For the synthesis of **3k**, proline *tert*-butyl ester was not compatible with the developed reaction under normal conditions, and we thought that this might be due to the additional steric hindrance around the secondary proline amine making it too poor a nucleophile to successfully intercept the acyl palladium species formed during the reaction. Therefore, we attempted the addition of a catalytic amount of 4-dimethylaminopyridine (DMAP) to in situ form the more electrophilic acylpyridinium species.^35^ This proved to work better with the demanding proline substrate, and product **3k** could be isolated in 40% yield. The lysine methyl ester was used in the commercially available Boc-protected form to provide substrate **3l** in an 87% isolated yield.

Possible racemization of the α-position of the amino acid in the optimized aminocarbonylation protocol was examined by comparing the specific rotation of products **3a** and **3e** with optically pure samples, where no racemization was detected for **3a**, and a 90% ee was determined for **3e**. Compounds **3a** and **3f** were also submitted for SFC-UV enantiomeric ratio analysis; however, partial transesterification to the corresponding methyl esters caused by the methanol cosolvent made the determination of the enantiomeric ratios difficult.

Due to this, the methyl ester analogues of **3a** and **3f** were prepared through our optimized method, and the enantiomeric ratio was analyzed through SFC chromatographic separation of the enantiomers and detection with PDA. The enantiomeric ratio was confirmed to be >99:1 upon analysis of the phenylalanine analog, while the phenylglycine analogue was found to have racemized under the reaction conditions (see the Supporting Information).

**Scheme 2:**
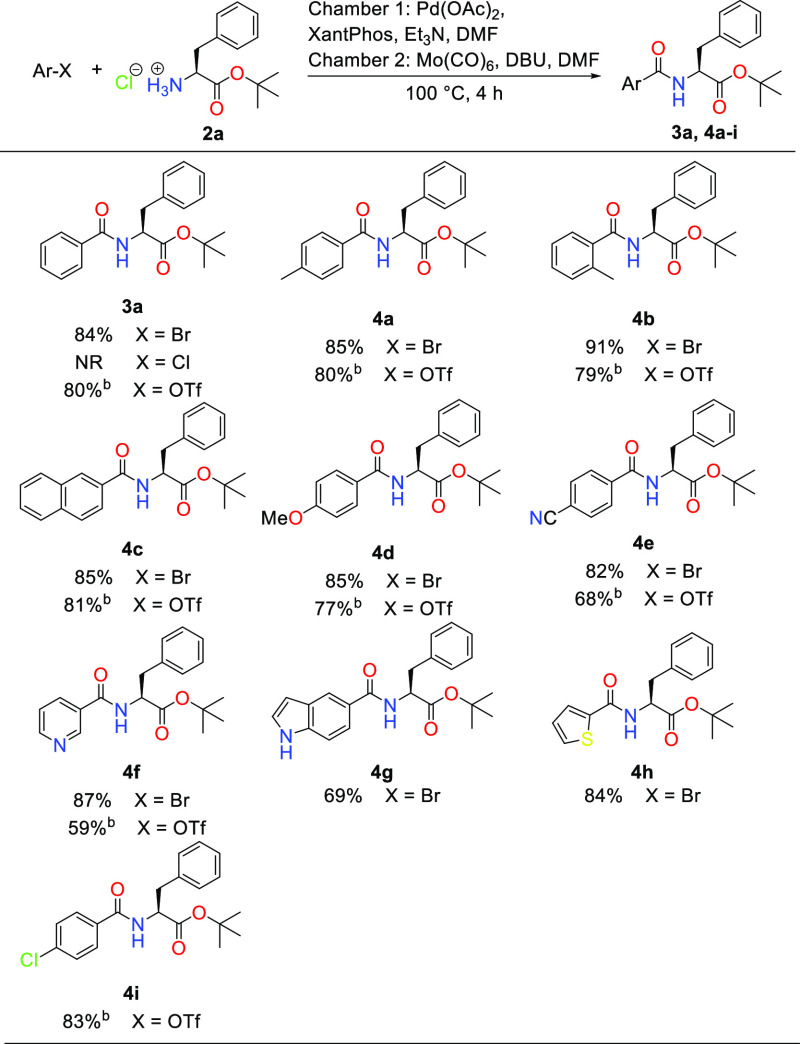
Substrate Scope with Regards to Aryl Halide or Triflate Reaction conditions for **4a**–**4m**: Chamber 1: Aryl bromide or triflate (1 equiv), phenylalanine *tert*-butyl ester hydrochloride (1.2 equiv), Pd(OAc)_2_ (1 mol %), XantPhos (2 mol %), triethylamine (3 equiv), and DMF. Chamber 2: Mo(CO)_6_ (2 equiv), DBU (3 equiv), and DMF. Isolated yields. For aryl triflates reactions were carried out with the addition of 10 mol % LiBr. NR = no reaction.

Next, we examined the scope of the aryl halide used in the aminocarbonylation reaction (Scheme 2). Exchanging the bromide for the much less reactive chloride provided no reactivity under the optimized conditions. Benzyl bromide also proved challenging, with only trace amounts of product formed after 4 h, and none could be isolated. The toluene derivatives **4a** and **4b** accommodated well within our conditions and provided 85 and 91% isolated yields, respectively. The introduction of more steric hindrance around the bromide in the form of 2-bromo-*m*-xylene proved detrimental to the reaction, where only trace amounts of the product could be detected by LC–UV/MS analysis (results not shown). Earlier reports have indicated that the two methyl groups of 2-bromo-*m*-xylene present a steric hindrance to ligand substitution by carbon monoxide in the axial coordination sites of the palladium complex, thus strongly inhibiting the reaction.^36^ The naphthyl-derivative **4c** was synthesized in 85% yield; however, the more sterically demanding 9-bromoanthracene and 1-bromonaphthalene were not compatible with the reaction. The aryl bromides bearing an electron-rich methoxy or electron-withdrawing nitrile substituent at the para position both reacted smoothly under the optimized conditions and offered the corresponding products in 85 (**4d**) and 82% (**4e**) yield. Furthermore, heteroaryl bromides such as 3-bromopyridine and 2-bromothiophene were well accepted in the aminocarbonylation, and the products were obtained in good yield (87% for **4f** and 84% for **4h**). 5-Bromoindole provides the corresponding product **4g** in a slightly lower yield (69%).

Then, phenyl triflates as readily available starting materials were investigated under the same reaction conditions, and the desired product **4a** was isolated in a relatively low 40% yield. The addition of halide salts to cross-coupling reactions involving aryl triflates, effectively replacing the weakly coordinating triflate in the postoxidative addition palladium(II)-complex with a halide ion, has been reported.^37,38^ Halide salts have also been found to stabilize palladium(0) species^39^ and were therefore trialed with our optimized conditions. We were delighted to see that the addition of 10 mol % lithium bromide increased the yield of product **3a** from 40 to 80%. Applying this new modified method to the toluene derivatives **4a** and the more sterically hindered **4b** gratifyingly produced similar yields. Bulky aryl triflates such as 2-naphthyl triflate were found to be compatible with the reaction conditions and yielded 81% of the desired product **4c**. In the case of 1-naphthyl triflate, similarly to when using 1-naphthyl bromide, only starting material was recovered on flash chromatography with no product-containing fractions. The results motivated us to investigate other aryl triflates possessing methoxy and nitrile substituents that delivered desired products **4d** and **4e** in good yield (77 and 68%, respectively). Moreover, heteroaryl triflates such as 3-pyridyl triflate were also compatible with this aminocarbonylation and offered product **4f** in 59% yield. Notably, 4-chlorophenyl triflate reacts chemoselectively with l-phenylalanine *tert*-butyl ester hydrochloride to offer desired product **4i** in 83% yield.

## Conclusions

In summary, we have developed a palladium-catalyzed two-chamber aminocarbonylation reaction for the N-capping of amino acids with aromatics and heteroaromatics. The method has a wide scope for both the amino acid and (hetero)aryl bromide coupling partners, and we have also demonstrated the possibility of extending the substrate scope to include various aryl and heteroaryl triflates.

## Experimental Section

### General Methods and Instrumentation

^1^H- and ^13^C-spectra were recorded on a Bruker 400 MHz instrument with chemical shifts reported in parts per million (ppm) and using the residual solvent peak as an internal reference. High-resolution mass spectra (HRMS) were recorded using a mass spectrometer with electrospray ionization with a 7-T hybrid linear ion trap. Thin-layer chromatography (TLC) with Supelco TLC plates with fluorescence indicator (254 nm) as well as liquid chromatography–mass spectrometry (LC/MS) on a Thermo Fischer Scientific UltiMate 3000 HPLC system with an MSQ Plus mass spectrometer was used for monitoring of reactions. Manual flash chromatography was run using silica gel (230–400 mesh). Automated flash chromatography was conducted on a Biotage Isolera One flash purification instrument with Biotage Sfär cartridges. For heating of reactions, DrySyn plates were used as heating mantle. Optical rotation was recorded on a Rudolph Autopol II polarimeter. Palladium-catalyzed carbonylation reactions were run in two fused 5 mL microwave vials (H-tubes).

### Method for Synthesis of Compounds with Aryl Halide Starting Material (Schemes 1 and 2)

Palladium acetate (1.4 mg, 6.4 μmol, 1 mol %), XantPhos (7.4 mg, 13 μmol, 2 mol %), amino acid ester hydrochloride (1.2 equiv), aryl halide (1 equiv, 0.64 mmol), and triethylamine (270 μL, 1.9 mmol, 3 equiv) were added to the first chamber of an H-tube. Molybdenum hexacarbonyl (340 mg, 1.3 mmol, 2 equiv) was weighed into the second chamber, and both chambers were sealed. Dry DMF (2 mL) was added to both chambers, and DBU (290 μL, 1.9 mmol, 3 equiv) was added to the molybdenum hexacarbonyl-containing chamber. The solutions were held at 100 °C for 4 h. After the solutions had cooled, the vials were vented to remove excess carbon monoxide, and 40 mL of EtOAc was added. DMF was removed by washing with water (3 × 30 mL), followed by brine (30 mL), and the organic phase was dried with MgSO_4_, filtered, and evaporated to yield the crude product, which was isolated by flash chromatography.

### Method for Synthesis of Aryl Triflates

Aryl triflates used for the preparation of **4e**, **4f**, and **4i** in Scheme 2 were synthesized according to a previously published procedure.^40^

### Method for Synthesis of Compounds with Aryl Triflate Starting Material (Scheme 2)

Palladium acetate (1.0 mg, 4.4 μmol, 1 mol %), XantPhos (5.1 mg, 8.8 μmol, 2 mol %), amino acid ester hydrochloride (1.2 equiv), aryl triflate (1 equiv, 0.44 mmol), triethylamine (190 μL, 1.3 mmol, 3 equiv), and lithium bromide (4.2 mg, 49 μmol, 10 mol %) were added to the first chamber of an H-tube. Molybdenum hexacarbonyl (230 mg, 0.88 mmol, 2 equiv) was weighed into the second chamber, and both chambers were sealed. Dry DMF (2 mL) was added to both chambers, and DBU (200 μL, 1.3 mmol, 3 equiv) was added to the molybdenum hexacarbonyl-containing chamber. The solutions were heated at 100 °C for 4 h. After the solutions had cooled, the vials were vented to remove excess carbon monoxide, and 40 mL of EtOAc was added. DMF was removed by washing with water (3 × 30 mL), followed by brine (30 mL), and the organic phase was dried with MgSO_4_, filtered, and evaporated to yield the crude product, which was isolated by flash chromatography.

#### *tert*-Butyl Benzoyl-l-phenylalaninate (**3a**)

Compound **3a** was purified through flash chromatography (15–20% EtOAc in *i*-hexane) to provide the product as a white solid in 84% (190 mg) isolated yield using bromobenzene as the starting material. The reaction was also conducted on a 1.5 mmol scale, following the same general procedure outlined for bromobenzene as starting material, providing the product with 78% (380 mg) isolated yield.

Using phenyl trifluoromethanesulfonate as starting material, the product was isolated in 80% (115 mg) yield. ^1^H NMR (400 MHz, chloroform-*d*) δ 7.71–7.62 (m, 2H), 7.45–7.37 (m, 1H), 7.33 (m, 2H), 7.24–7.08 (m, 5H), 6.60 (d, *J* = 7.5 Hz, 1H), 4.88 (ddd, *J* = 7.5, 6.1, 5.4 Hz, 1H), 3.22–3.10 (m, 2H), 1.36 (s, 9H). ^13^C{^1^H} NMR (101 MHz, chloroform-*d*) δ: 170.9, 166.8, 136.3, 134.2, 131.8, 129.7, 128.7, 128.5, 127.10, 127.08, 82.7, 54.0, 38.1, 28.1. HRMS: calcd for C_20_H_24_NO_3_ [M + H]+ 326.1756; found: 326.1757. Opt. rot.: [α]_589_^25^ = +13.6 (*c* = 1.03, THF).

#### Methyl Benzoyl-l-alaninate (**3b**)

Compound **3b** was purified through flash chromatography (15–25% EtOAc in *i*-hexane) to provide the product as a white solid in 61% (79.3 mg) isolated yield using bromobenzene as the starting material. ^1^H NMR (400 MHz, chloroform-d) δ 7.86–7.75 (m, 2H), 7.53–7.47 (m, 1H), 7.47–7.39 (m, 2H), 6.78 (d, *J* = 7.4 Hz, 1H), 4.80 (dq, *J* = 7.4, 7.1 Hz, 1H), 3.78 (s, 3H), 1.52 (d, *J* = 7.1 Hz, 3H). ^13^C{^1^H} NMR (101 MHz, chloroform-*d*) δ: 173.8, 166.9, 134.0, 131.9, 128.7, 127.2, 52.7, 48.6, 18.8. HRMS: calcd for C_11_H_14_NO_3_ [M + H]+ 208.0974; found: 208.0983. Opt. rot.: [α]_589_^25^ = +8.00 (*c* = 0.10, THF).

#### Methyl Benzoyl-l-valinate (**3c**)

Compound **3c** was purified through flash chromatography (15–20% EtOAc in *i*-hexane) to provide the product as a white solid in 80% (126 mg) isolated yield using bromobenzene as starting material. ^1^H NMR (400 MHz, chloroform-d) δ 7.85–7.77 (m, 2H), 7.55–7.48 (m, 1H), 7.48–7.41 (m, 2H), 6.63 (d, *J* = 8.6 Hz, 1H), 4.79 (dd, *J* = 8.6, 4.9 Hz, 1H), 3.77 (s, 3H), 2.35–2.21 (m, 1H), 1.01 (d, *J* = 6.9 Hz, 3H), 0.99 (d, *J* = 6.9 Hz, 3H). ^13^C{^1^H} NMR (101 MHz, chloroform-d) δ: 172.8, 167.4, 134.3, 131.9, 128.8, 127.2, 57.5, 52.4, 31.8, 19.1, 18.1. HRMS: calcd for C_13_H_18_NO_3_ [M + H]+ 236.1287; found: 236.1276. Opt. rot.: [α]_589_^25^ = +9.62 (*c* = 0.10, THF).

#### Methyl Benzoyl-l -isoleucinate (**3d**)

Compound **3d** was purified through flash chromatography (15–20% EtOAc in *i*-hexane) to provide the product as a white solid in 88% (146 mg) isolated yield using bromobenzene as starting material. ^1^H NMR (400 MHz, chloroform-d) δ 7.83–7.77 (m, 2H), 7.54–7.48 (m, 1H), 7.48–7.40 (m, 2H), 6.66 (d, *J* = 8.5 Hz, 1H), 4.82 (dd, *J* = 8.5, 5.0 Hz, 1H), 3.77 (s, 3H), 2.08–1.95 (m, 1H), 1.59–1.47 (m, 1H), 1.33–1.19 (m, 1H), 1.00–0.93 (m, 6H). ^13^C{^1^H} NMR (101 MHz, chloroform-*d*) δ: 172.8, 167.2, 134.3, 131.9, 128.7, 127.2, 56.9, 52.3, 38.4, 25.5, 15.6, 11.8. HRMS: calcd for C_14_H_20_NO_3_ [M + H]+ 250.1443; found: 250.1432. Opt. rot.: [α]_589_^25^ = +15.2 (*c* = 0.10, THF).

#### Methyl Benzoyl-l-leucinate (**3e**)

Compound **3e** was purified through flash chromatography (15–20% EtOAc in *i*-hexane) to provide the product as a white solid in 84% (134 mg) isolated yield using an aryl bromide starting material. ^1^H NMR (400 MHz, chloroform-d) δ 7.86–7.75 (m, 2H), 7.54–7.47 (m, 1H), 7.47–7.38 (m, 2H), 6.57 (d, *J* = 8.4 Hz, 1H), 4.95–4.80 (m, 1H), 3.77 (s, 3H), 1.82–1.61 (m, 3H), 1.04–0.94 (m, 6H). ^13^C{^1^H} NMR (101 MHz, chloroform-*d*) δ: 173.8, 167.2, 134.1, 131.9, 128.7, 127.2, 52.5, 51.3, 42.0, 25.1, 23.0, 22.2. HRMS: calcd for C_14_H_20_NO_3_ [M + H]+ 250.1443; found: 250.1431. Opt. rot.: [α]_589_^25^ = −8.90 (*c* = 0.74, THF).

#### Ethyl (*R*)-2-Benzamido-2-phenylacetate (**3f**)

Compound **3f** was purified through flash chromatography (15–20% EtOAc in *i*-hexane) to provide the product as a white solid in 87% (158 mg) isolated yield using bromobenzene as starting material. ^1^H NMR (400 MHz, chloroform-d) δ 7.87–7.79 (m, 2H), 7.51 (m, 1H), 7.48–7.40 (m, 4H), 7.40–7.30 (m, 3H), 7.18 (d, *J* = 7.0 Hz, 1H), 5.77 (d, *J* = 7.0 Hz, 1H), 4.33–4.13 (dm, 2H), 1.24 (t, *J* = 7.1 Hz, 3H). ^13^C{^1^H} NMR (101 MHz, chloroform-*d*) δ: 171.2, 166.6, 136.9, 133.8, 132.0, 129.1, 128.7, 128.6, 127.4, 127.3, 62.2, 57.0, 14.2. HRMS: calcd for C_17_H_18_NO_3_ [M + H]+ 284.1287; found: 284.1273. Opt. rot.: [α]_589_^25^ = +4.72 (*c* = 0.11, THF).

#### Methyl (2*S*)-2-Benzamido-3-(4-*tert*-butoxyphenyl)propanoate (**3g**)

Compound **3g** was purified through flash chromatography (15–30% EtOAc in *i*-hexane) to provide the product as a white solid in 87% (206 mg) isolated yield using bromobenzene as starting material. ^1^H NMR (400 MHz, chloroform-d) δ 7.74–7.67 (m, 2H), 7.53–7.46 (m, 1H), 7.45–7.37 (m, 2H), 7.06–6.99 (m, 2H), 6.95–6.88 (m, 2H), 6.54 (d, *J* = 7.5 Hz, 1H), 5.05 (dt, *J* = 7.5, 5.8 Hz, 1H), 3.74 (s, 3H), 3.24 (dd, *J* = 13.9, 5.8 Hz, 1H), 3.18 (dd, *J* = 13.9, 5.8 Hz, 1H), 1.32 (s, 9H). ^13^C{^1^H} NMR (101 MHz, chloroform-*d*) δ: 172.2, 166.9, 154.7, 134.1, 131.9, 130.7, 129.9, 128.8, 127.1, 124.4, 78.6, 53.7, 52.5, 37.4, 29.0. HRMS: calcd for C_21_H_26_NO_4_ [M + H]+ 356.1862; found: 356.1860. Opt. rot.: [α]_589_^25^ = −2.66 (*c* = 0.11, THF).

#### Methyl *N*-Benzoyl-*O*-(*tert*-butyl)-l-serinate (**3h**)

Compound **3h** was purified through flash chromatography (15–30% EtOAc in *i*-hexane) to provide the product as a noncolored oil in 85% (151 mg) isolated yield using bromobenzene as starting material. ^1^H NMR (400 MHz, chloroform-d) δ 7.86–7.77 (m, 2H), 7.55–7.48 (m, 1H), 7.47–7.39 (m, 2H), 6.97 (d, *J* = 8.3 Hz, 1H), 4.92 (ddd, *J* = 8.3, 3.2, 3.0 Hz, 1H), 3.90 (dd, *J* = 9.1, 3.0 Hz, 1H), 3.77 (s, 3H), 3.69 (dd, *J* = 9.1, 3.2 Hz, 1H), 1.14 (s, 9H). ^13^C{^1^H} NMR (101 MHz, chloroform-*d*) δ: 171.2, 167.2, 134.2, 131.8, 128.7, 127.2, 73.6, 62.2, 53.4, 52.5, 27.4. HRMS: calcd for C_15_H_22_NO_4_ [M + H]+ 280.1549; found: 280.1538. Opt. rot.: [α]_589_^25^ = +46.82 (*c* = 0.19, THF).

#### Methyl *N*-Benzoyl-*O*-(*tert*-butyl)-l-threoninate (**3i**)

Compound **3i** was purified through flash chromatography (15–20% EtOAc in *i*-hexane) to provide the product as a noncolored oil in 88% (161 mg) isolated yield using bromobenzene as starting material. ^1^H NMR (400 MHz, chloroform-d) δ 7.89–7.80 (m, 2H), 7.55–7.49 (m, 1H), 7.49–7.41 (m, 2H), 6.89 (d, *J* = 9.2 Hz, 1H), 4.72 (dd, *J* = 9.2, 1.9 Hz, 1H), 4.32 (qd, *J* = 6.3, 1.9 Hz, 1H), 3.73 (s, 3H), 1.25 (d, *J* = 6.3 Hz, 3H), 1.15 (s, 9H). ^13^C{^1^H} NMR (101 MHz, chloroform-*d*) δ: 171.5, 167.9, 134.4, 131.8, 128.7, 127.3, 74.3, 67.8, 58.3, 52.4, 28.5, 21.3. HRMS: calcd for C_16_H_24_NO_4_ [M + H]+ 294.1705; found: 294.1700. Opt. rot.: [α]_589_^25^ = +53.45 (*c* = 0.13, THF).

#### 5-(*tert*-Butyl) 1-Methyl Benzoyl-l-glutamate (**3j**)

Compound **3j** was purified through flash chromatography (15–30% EtOAc in *i*-hexane) to provide the product as a noncolored oil in 66% (135 mg) isolated yield using bromobenzene as starting material. ^1^H NMR (400 MHz, chloroform-d) δ 7.85–7.79 (m, 2H), 7.53–7.46 (m, 1H), 7.45–7.38 (m, 2H), 7.15 (d, *J* = 7.4 Hz, 1H), 4.76 (ddd, *J* = 8.2, 7.4, 4.8 Hz, 1H), 3.76 (s, 3H), 2.49–2.29 (m, 2H), 2.29–2.17 (m, 1H), 2.15–2.04 (m, 1H), 1.41 (s, 9H). ^13^C{^1^H} NMR (101 MHz, chloroform-*d*) δ: 172.8, 172.7, 133.7, 131.9, 128.6, 127.3, 81.2, 52.7, 52.6, 31.7, 28.1, 27.1. HRMS: calcd for C_17_H_24_NO_5_ [M + H]+ 322.1654; found: 322.1640. Opt. rot.: [α]_589_^25^ = −3.68 (*c* = 0.14, THF).

#### *tert*-Butyl Benzoyl-l-prolinate (**3k**)

Palladium acetate (1.4 mg, 6.4 μmol, 1 mol %), XantPhos (7.4 mg, 13 μmol, 2 mol %), *tert*-butyl (2*S*)-pyrrolidine-2-carboxylate (130, 0.76 mmol, 1.2 equiv), bromobenzene (100 mg, 0.64 mmol, 1 equiv), triethylamine (270 μL, 1.9 mmol, 3 equiv), and a catalytic amount of DMAP was added to the first chamber of an H-tube. Molybdenum hexacarbonyl (340 mg, 1.3 mmol, 2 equiv) was weighed into the second chamber, and both chambers were sealed. Dry DMF (2 mL) was added to both chambers, and DBU (280 μL, 1.9 mmol, 3 equiv) was added to the molybdenum hexacarbonyl-containing chamber. The solutions were heated at 100 °C for 4 h. After the solutions had cooled, the vials were vented to remove excess carbon monoxide, and 40 mL of EtOAc was added. DMF was removed by washing with water (3 × 30 mL), followed by brine (30 mL), and the organic phase was dried with MgSO4, filtered, and evaporated to yield the crude product, which was isolated by flash chromatography (15–30% EtOAc in *i*-hexane) to provide the product **3k** as a white solid in 40% (70 mg) isolated yield. The product was a mixture between rotamers at a 7:3 ratio at the conditions used for NMR analysis.^41^^1^H NMR (400 MHz, methanol-d4) δ 7.56 (dd, *J* = 7.5, 2.0 Hz, 2H), 7.52–7.45 (m, 2H), 7.43 (d, *J* = 3.8 Hz, 1H), 4.48 (major; dd, *J* = 8.5, 5.0 Hz, 1H), 4.38 (minor; dd, *J* = 8.8, 2.8 Hz, 1H), 3.80–3.66 (minor; m, 2H), 3.65–3.50 (major; m, 2H), 2.44–2.27 (minor; m, 4H), 2.07–1.85 (major; m, 4H), 1.52 (major; s, 9H), 1.29 (minor; s,9H). ^13^C{^1^H} NMR (101 MHz, chloroform-d) δ: 171.4, 170.5 (minor), 169.5 (major), 137.1 (minor), 136.5 (major), 130.0 (major), 129.7 (minor), 128.3 (minor), 128.2 (major), 127.2 (major), 126.8 (minor), 81.7 (minor), 81.3 (major), 62.0 (minor), 60.0 (major), 50.0 (major), 46.7 (minor), 31.6 (minor), 29.4 (major), 28.0 (major), 27.7 (minor), 25.4 (major), 22.6 (minor). HRMS: calcd for C_16_H_22_NO_3_ [M + H]+ 276.1594; found: 276.1607. Opt. rot.: [α]_589_^25^ = −60.02 (*c* = 0.15, THF).

#### Methyl *N*^2^-Benzoyl-*N*^6^-(*tert*-butoxycarbonyl)-l-lysinate (**3l**)

Compound **3l** was purified through flash chromatography (30–50% EtOAc in *i*-hexane) to provide the product as a white solid in 87% (203 mg) isolated yield using bromobenzene as starting material. ^1^H NMR (400 MHz, chloroform-d) δ 7.85–7.78 (m, 2H), 7.54–7.47 (m, 1H), 7.47–7.40 (m, 2H), 6.77 (d, *J* = 7.7 Hz, 1H), 4.86–4.75 (m, 1H), 4.61 (s, 1H), 3.77 (s, 3H), 3.16–3.05 (m, 2H), 2.03–1.91 (m, 1H), 1.87–1.75 (m, 1H), 1.61–1.31 (m, 13H). ^13^C{^1^H} NMR (101 MHz, chloroform-*d*) δ: 173.2, 167.3, 156.2, 134.0, 131.9, 128.7, 127.3, 79.2, 52.6, 52.5, 40.2, 32.4, 29.7, 28.5, 22.6. HRMS: calcd for C_19_H_29_N_2_O_5_ [M + H]+ 365.2076; found: 365.2063. Opt. rot.: [α]_589_^25^ = −3.54 (*c* = 0.11, THF).

#### Methyl (4-Methylbenzoyl)-l-phenylalaninate (**4a**)

Compound **4a** was purified through flash chromatography (10–15% EtOAc in *i*-hexane) to provide the product as a white solid in 85% (221 mg) isolated yield using 1-bromo-4-methylbenzene as starting material. Using *p*-tolyl trifluoromethanesulfonate as starting material, the product was isolated in 80% (113 mg) yield. ^1^H NMR (400 MHz, chloroform-d) δ 7.68–7.61 (m, 2H), 7.30–7.16 (m, 7H), 6.62 (d, *J* = 7.1 Hz, 1H), 4.99–4.93 (m, 1H), 3.27–3.20 (m, 2H), 2.39 (s, 3H), 1.44 (s, 9H). ^13^C{^1^H} NMR (101 MHz, chloroform-d) δ: 170.9, 166.7, 142.2, 136.4, 131.4, 129.8, 129.4, 128.5, 127.1, 127.1, 82.7, 54.0, 38.2, 28.1, 21.6. HRMS: calcd for C_21_H_26_NO_3_ [M + H]+ 340.1907; found: 340.1913. Opt. rot.: [α]_589_^25^ = +15.93 (*c* = 0.15, THF).

#### *tert*-Butyl (2-Methylbenzoyl)-l -phenylalaninate (**4b**)

Compound **4b** was purified through flash chromatography (15–25% EtOAc in *i*-hexane) to provide the product as a white solid in 91% (181 mg) isolated yield using 1-bromo-2-methylbenzene as starting material. Using *o*-tolyl trifluoromethanesulfonate as starting material, the product was isolated in 79% (110 mg) yield. ^1^H NMR (400 MHz, chloroform-d) δ 7.32–7.30 (m, 1H), 7.30–7.28 (m, 2H), 7.28–7.27 (m, 1H), 7.26–7.23 (m, 1H), 7.23–7.21 (m, 1H), 7.21–7.14 (m, 3H), 6.22 (d, *J* = 7.8 Hz, 1H), 4.95 (ddd, *J* = 7.8, 6.2, 6.0 Hz, 1H), 3.27 (dd, *J* = 13.9, 6.2 Hz, 1H), 3.17 (dd, *J* = 13.9, 6.0 Hz, 1H), 2.39 (s, 3H), 1.45 (s, 9H). ^13^C{^1^H} NMR (101 MHz, chloroform-d) δ: 170.8, 169.4, 136.5, 136.4, 135.9, 131.2, 130.2, 129.7, 128.6, 127.1, 127.0, 125.8, 82.6, 53.9, 38.2, 28.1, 19.9. HRMS: calcd for C_21_H_26_NO_3_ [M + H]+ 340.1907; found: 340.1913. Opt. rot.: [α]_589_^25^ = −5.88 (*c* = 0.101, THF).

#### Methyl (2-Naphthoyl)-l-phenylalaninate (**4c**)

Compound **4c** was purified through flash chromatography (15–20% EtOAc in *i*-hexane) to provide the product as a white solid in 85% (206 mg) isolated yield using 2-bromonaphthalene as starting material. Using naphthalen-2-yl trifluoromethanesulfonate as starting material, the product was isolated in 81% (110 mg) yield. ^1^H NMR (400 MHz, chloroform-d) δ 8.26 (s, 1H), 7.94–7.85 (m, 3H), 7.81 (dd, *J* = 8.5, 1.8 Hz, 1H), 7.60–7.51 (m, 2H), 7.32–7.26 (m, 3H), 7.25–7.17 (m, 2H), 6.79 (d, *J* = 7.3 Hz, 1H), 5.07–4.99 (m, 1H), 3.34–3.22 (m, 1H), 1.46 (s, 9H). ^13^C{^1^H} NMR (101 MHz, chloroform-*d*) δ: 171.0, 166.8, 136.4, 135.0, 132.7, 131.5, 129.8, 129.1, 128.6, 128.5, 127.89, 127.86, 127.7, 127.2, 126.9, 123.7, 82.8, 54.1, 38.2, 28.2. HRMS: calcd for C_24_H_26_NO_3_ [M + H]+ 376.1907; found: 376.1913.. Opt. rot.: [α]_589_^25^ = +5.26 (*c* = 0.095, THF).

#### *tert*-Butyl (4-Methoxybenzoyl)-l-phenylalaninate (**4d**)

Compound **4d** was purified through flash chromatography (20–30% EtOAc in *i*-hexane) to provide the product as a white solid in 85% (162 mg) isolated yield using 1-bromo-4-methoxybenzene as starting material. Using 4-methoxyphenyl trifluoromethanesulfonate as starting material, the product was isolated in 77% (105 mg) yield. ^1^H NMR (400 MHz, chloroform-d) δ 7.74–7.68 (m, 2H), 7.30–7.27 (m, 1H), 7.26–7.20 (m, 2H), 7.20–7.18 (m, 1H), 7.18–7.16 (m, 1H), 6.91 (m, 2H), 6.56 (s, 1H), 4.95 (dt, *J* = 7.4, 5.7 Hz, 1H), 3.84 (s, 2H), 3.22 (d, *J* = 5.7 Hz, 2H), 1.43 (s, 9H). ^13^C{^1^H} NMR (101 MHz, chloroform-d): 171.0, 166.3, 162.5, 136.4, 129.8, 128.9, 128.5, 127.1, 126.6, 113.9, 82.7, 55.5, 38.2, 28.1. HRMS: calcd for C_21_H_26_NO_4_ [M + H]+ 356.1856; found: 356.1862. Opt. rot.: [α]_589_^25^ = +13.21 (*c* = 0.159, THF).

#### *tert*-Butyl (4-Cyanobenzoyl)-l-phenylalaninate (**4e**)

Compound **4e** was purified through flash chromatography (25–35% EtOAc in *i*-hexane) to provide the product as a white solid in 82% (158 mg) isolated yield using 4-bromobenzonitrile as starting material. Using 4-cyanophenyl trifluoromethanesulfonate as starting material, the product was isolated in 68% (95.0 mg) yield. ^1^H NMR (400 MHz, chloroform-d) δ 8.94–8.91 (m, 1H), 8.72 (dd, *J* = 4.8, 1.6 Hz, 1H), 8.05 (dt, *J* = 7.9, 2.0 Hz, 1H), 7.39–7.34 (m, 1H), 7.32–7.26 (m, 2H), 7.26–7.21 (m, 1H), 7.20–7.15 (m, 2H), 6.68 (d, *J* = 7.4 Hz, 1H), 4.95 (ddd, *J* = 7.4, 6.1, 5.5 Hz, 1H), 3.30–3.18 (m, 2H), 1.45 (s, 9H). ^13^C{^1^H} NMR (101 MHz, chloroform-d) δ: 170.7, 165.0, 138.1, 136.0, 132.6, 129.6, 128.6, 127.8, 127.3, 118.2, 115.4, 83.2, 54.2, 37.9, 28.1. HRMS: calcd for C_21_H_23_N_2_O_3_ [M + H]+ 351.1703; found: 351.1717. Opt. rot.: [α]_589_^25^ = +9.31 (*c* = 0.172, THF).

#### *tert*-Butyl Nicotinoyl-l-phenylalaninate (**4f**)

Compound **4f** was purified through flash chromatography (30–50% EtOAc in *i*-hexane) to provide the product as a noncolored oil in 87% (180 mg) isolated yield using 3-bromopyridine as starting material. Using pyridin-3-yl trifluoromethanesulfonate as starting material, the product was isolated in 59% (84.0 mg) yield. ^1^H NMR (400 MHz, chloroform-d) δ 8.96–8.89 (m, 1H), 8.72 (dd, *J* = 4.8, 1.6 Hz, 1H), 8.05 (dt, *J* = 7.9, 2.0 Hz, 1H), 7.36 (ddt, *J* = 8.0, 4.9, 0.8 Hz, 1H), 7.32–7.26 (m, 2H), 7.26–7.21 (m, 1H), 7.20–7.15 (m, 2H), 6.68 (d, *J* = 7.4 Hz, 1H), 4.95 (dt, *J* = 7.4, 6.1, 5.5 Hz, 1H), 3.24 (dd, *J* = 13.9, 6.1 Hz, 1H), 3.21 (dd, *J* = 13.9, 5.4 Hz, 1H), 1.45 (s, 9H). ^13^C{^1^H} NMR (101 MHz, chloroform-d) δ: 170.7, 165.0, 152.3, 148.2, 136.0, 135.0, 129.8, 129.5, 128.5, 127.1, 123.4, 82.8, 54.0, 37.9, 28.0. HRMS: calcd for C_19_H_23_N_2_O_3_ [M + H]+ 327.1703; found: 327.1706. Opt. rot.: [α]_589_^25^ = +6.23 (*c* = 0.241, THF).

#### *tert*-Butyl (1*H*-Indole-5-carbonyl)-l-phenylalaninate (**4g**)

Compound **4g** was purified through flash chromatography (20–70% EtOAc in *i*-hexane) to provide the product as a brown viscous oil in 69% (128 mg) isolated yield using 5-bromo-1*H*-indole as starting material. ^1^H NMR (400 MHz, chloroform-d) δ 8.42 (s, 1H), 8.08 (s, 1H), 7.63 (dd, *J* = 8.5, 1.5 Hz, 1H), 7.42–7.38 (m, 1H), 7.31–7.27 (m, 2H), 7.26–7.19 (m, 3H), 6.69 (d, *J* = 7.4 Hz, 1H), 6.63–6.60 (m, 1H), 5.01 (ddd, *J* = 7.4, 6.1, 5.3 Hz, 1H), 3.27 (dd, *J* = 13.9, 6.1 Hz, 1H), 3.25 (dd, *J* = 13.9, 5.3 Hz, 1H), 1.44 (s, 9H). ^13^C{^1^H} NMR (101 MHz, chloroform-d) δ: 171.2, 168.1, 137.9, 136.5, 129.8, 128.5, 127.7, 127.1, 125.94, 125.85, 121.1, 120.4, 111.2, 103.7, 82.6, 54.1, 38.3, 28.1. HRMS: calcd for C_22_H_25_N_2_O_3_ [M + H]+ 365.1860; found: 365.1863. Opt. rot.: [α]_589_^25^ = +20.17 (*c* = 0.124, THF).

#### *tert*-Butyl (Thiophene-2-carbonyl)-l-phenylalaninate (**4h**)

Compound **4h** was purified through flash chromatography (10–15% EtOAc in *i*-hexane) to provide the product as a white solid in 84% (170 mg) isolated yield using 2-bromothiophene as starting material. ^1^H NMR (400 MHz, chloroform-d) δ 7.50 (dd, *J* = 3.7, 0.9 Hz, 1H), 7.49–7.39 (m, 1H), 7.35–7.27 (m, 3H), 7.27–7.22 (m, 2H), 7.10–7.00 (m, 1H), 6.90–6.69 (m, 1H), 4.97 (dd, *J* = 7.6, 6.0 Hz, 1H), 3.24 (d, *J* = 6.0 Hz, 2H), 1.46 (s, 9H). ^13^C{^1^H} NMR (101 MHz, chloroform-d) δ: 171.1, 161.6, 138.8, 136.5, 130.6, 129.9, 128.7, 128.6, 127.9, 127.3, 82.9, 54.2, 38.4, 28.3. HRMS: calcd for C_18_H_22_NO_3_S [M + H]+ 332.1315; found: 332.1327. Opt. rot.: [α]_589_^25^ = +11.37 (*c* = 0.087, THF).

#### *tert*-Butyl (4-Chlorobenzoyl)-l-phenylalaninate (**4i**)

Compound **4i** was purified through flash chromatography (5–15% EtOAc in *i*-hexane) to provide the product as a white solid in 83% (115 mg) isolated yield using 4-chlorophenyl trifluoromethanesulfonate as starting material. ^1^H NMR (400 MHz, chloroform-d) δ 7.71–7.64 (m, 2H), 7.42–7.37 (m, 2H), 7.31–7.26 (m, 2H), 7.26–7.21 (m, 1H), 7.20–7.15 (m, 2H), 6.58 (d, *J* = 7.4 Hz, 1H), 4.93 (ddd, *J* = 7.4, 6.1, 5.3 Hz, 1H), 3.23 (dd, *J* = 13.9, 6.1 Hz, 1H), 3.22 (dd, *J* = 13.9, 5.4 Hz, 1H), 1.44 (s, 9H). ^13^C{^1^H} NMR (101 MHz, chloroform-d) δ: 170.8, 165.7, 138.0, 136.2, 132.6, 129.7, 129.0, 128.5, 127.2, 82.9, 54.0, 38.1, 28.1. HRMS: calcd for C_20_H_23_ClNO_3_ [M + H]+ 360.1361; found: 360.1360. Opt. rot.: [α]_589_^25^ = +11.00 (*c* = 0.101, THF).

## Data Availability

The data underlying this study are available in the published article and its Supporting Information.

## Supporting Information Available

Copies of all ^1^H- and ^13^C NMR spectra detailed in the experimental section and details regarding SFC chiral separation and analysis (PDF)
