# Supplementary material for: Two-Chamber Aminocarbonylation of Aryl Bromides and Triflates Using Amino Acids as Nucleophiles
Source: J Org Chem. 2023 Aug 28;88(18):12978–85. doi: 10.1021/acs.joc.3c00972 (PMC10507664; doi:10.1021/acs.joc.3c00972)
Supplement: Supplementary file 1 — jo3c00972_si_001.pdf [file jo3c00972_si_001.pdf]

Two-Chamber Aminocarbonylation of Aryl Bromides and Triflates Using Amino Acids as Nucleophiles

Supporting Information

Jens Lindman, Anubha Yadav, Johan Gising, Mats Larhed[*]

Mats.larhed@ilk.uu.se

Uppsala University, Department of Medicinal Chemistry, Husargatan 3, SE-751 23 Uppsala, Sweden

Contents

Determination of enantiomeric ratio 2

NMR-spectra of compounds **3a – 4i** 8

Determination of enantiomeric ratio

Methyl benzoyl-L-phenylalaninate

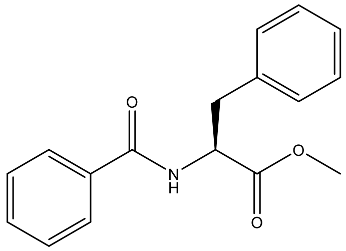

Methyl benzoyl-L-phenylalaninate was synthesized according to the general procedure outlined in the Experimental Section. The product was purified through flash chromatography (25- 30% EtOAc in i-hexane) to provide the product as a white solid in 84% isolated yield. ^{1}H NMR (400 MHz, Chloroform-*d*) δ 7.76 – 7.68 (m, 2H), 7.54 – 7.48 (m, 1H), 7.45 – 7.39 (m, 2H), 7.32 – 7.26 (m, 2H), 7.26 – 7.23 (m, 1H), 7.16 – 7.11 (m, 2H), 6.56 (d, *J* = 7.2 Hz, 1H), 5.10 (ddd, *J* = 7.2, 5.8, 5.4 Hz, 1H), 3.77 (s, 3H), 3.30 (dd, *J* = 13.8, 5.8 Hz, 1H), 3.23 (dd, *J* = 13.8, 5.4 Hz, 1H). ^{13}C NMR (101 MHz, Chloroform-*d*) δ 172.2, 166.9, 135.9, 134.0, 131.9, 129.5, 128.7 (2C), 127.3, 127.1, 53.6, 52.5, 38.0. HRMS: calcd. for $C_{17}H_{18}NO_3$ [M + H]+ 284.1287; found: 284.1292. Opt. rot.: $[\alpha]_{589}^{25} = +5.26$ (c = 1.01, THF).

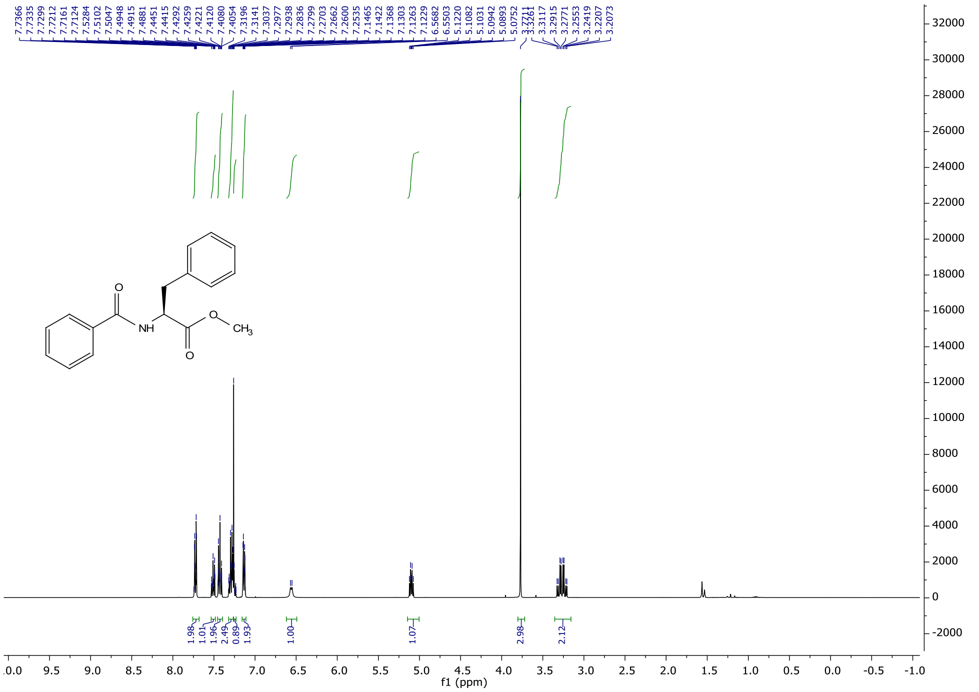

Figure S1. ^{1}H NMR (400 MHz, $CDCl_3$) spectra of methyl benzoyl-L-phenylalaninate.

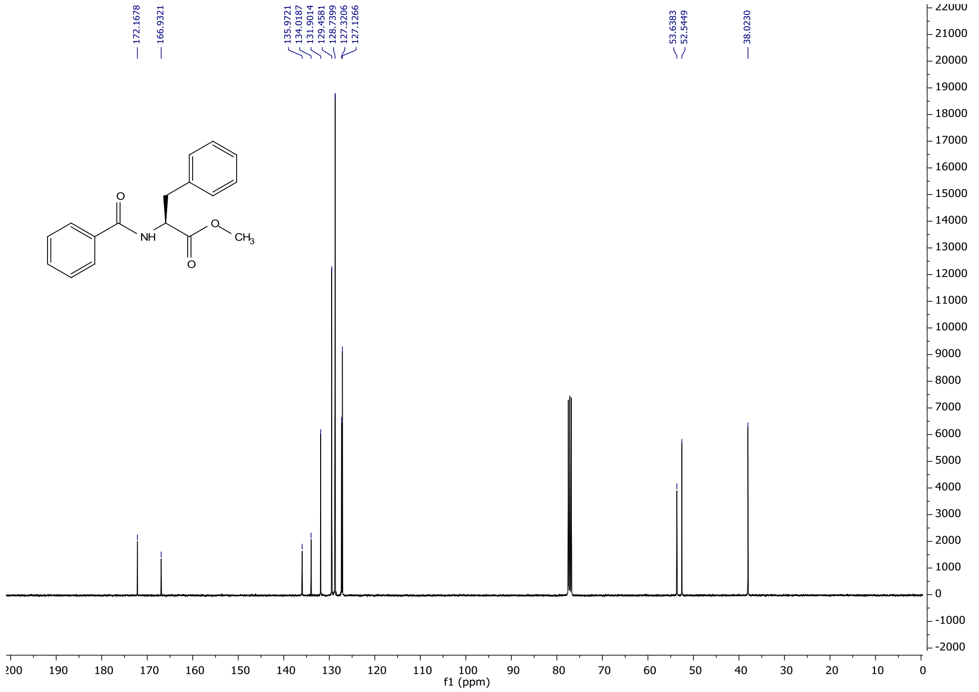

Figure S2. $^{13}C\{^{1}H\}$ NMR (101 MHz, $CDCl_3$) spectra of methyl benzoyl-L-phenylalaninate.

Determination of the enantiomeric ratio of methyl benzoyl-L-phenylalaninate was performed on a Supercritical Fluid Chromatography (SFC) system connected to a PDA detector. The results were compared to those of a racemic mixture analyzed using the same system. The samples were diluted to a concentration of around 1 mg/mL and 10 µL was injected on to a 5 µm, CHIRALPAK-IF, 4.6 mm × 150 mm (diameter x length) column held at 45 °C. A 5 min gradient of 5 – 50% MeOH in CO_2 followed by 3 min isocratic 50% MeOH in CO_2 was applied at a flow rate of 5 mL/min. The back pressure was set to 120 Bar. The PDA scanned from 220 to 400. The areas where the expected peaks from the two enantiomers would appear were integrated and the enantiomeric ratio was determined.

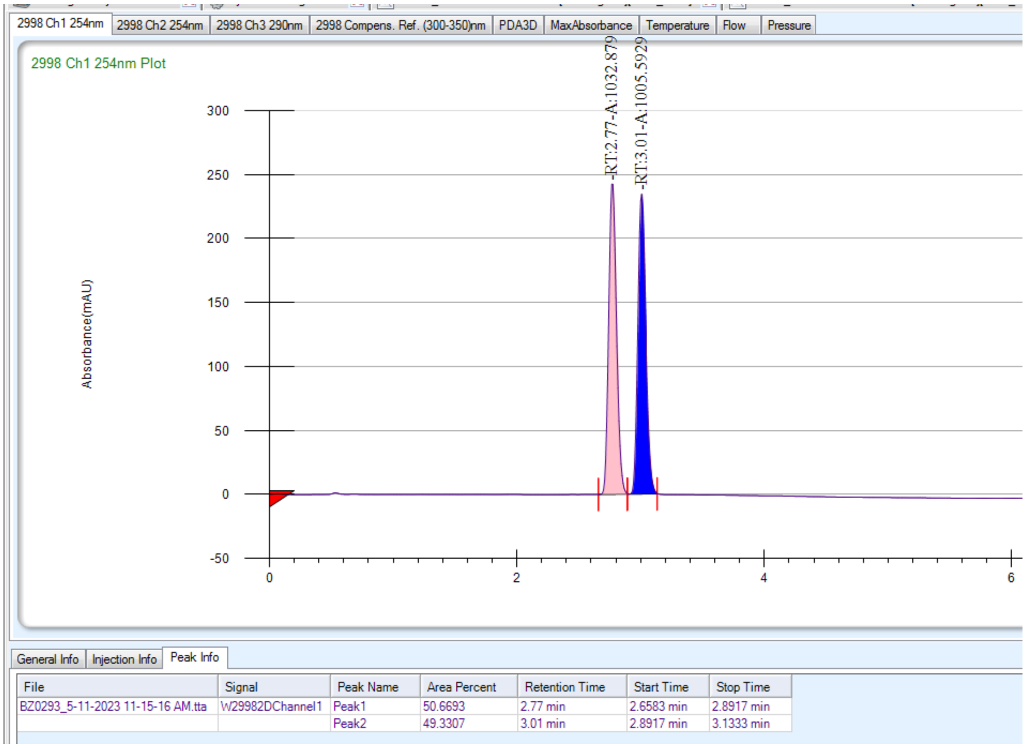

Figure S3. SFC-UV spectrum of racemic mixture of methyl benzoylphenylalaninate.

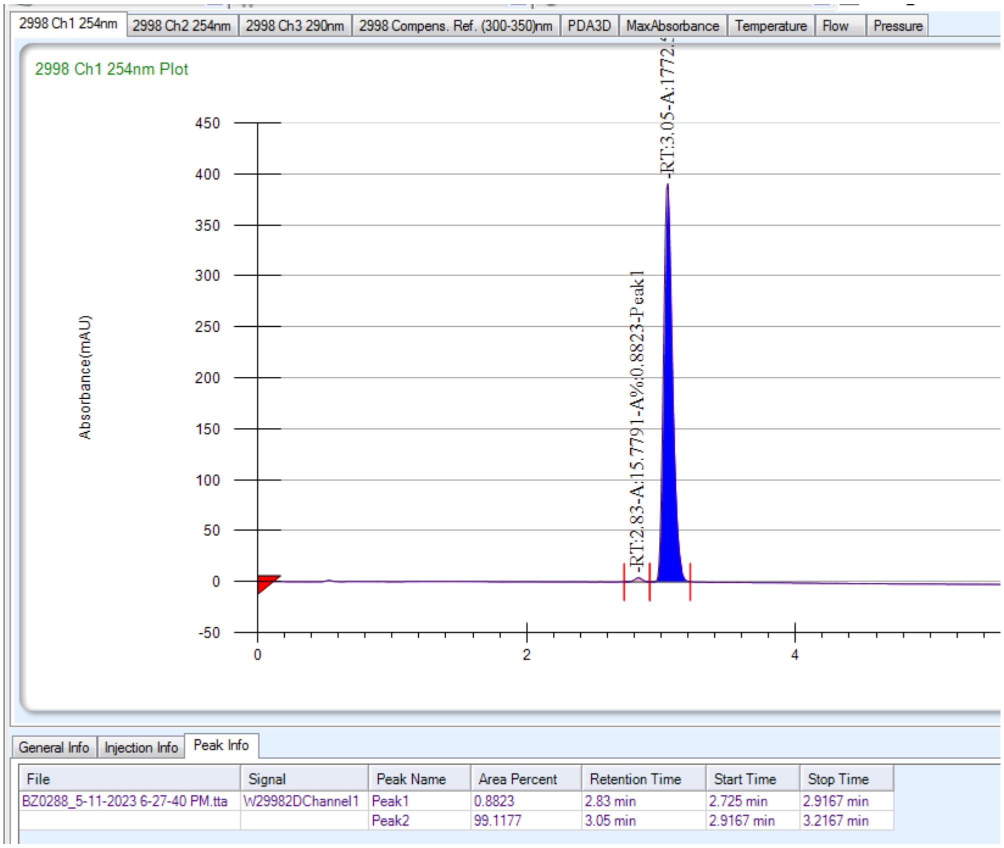

Figure S4. SFC-UV spectrum of methyl benzoyl-L-phenylalaninate synthesized through our optimized method displaying >99:1 enantiomeric ratio of the L-enantiomer.

Methyl (S)-2-benzamido-2-phenylacetate

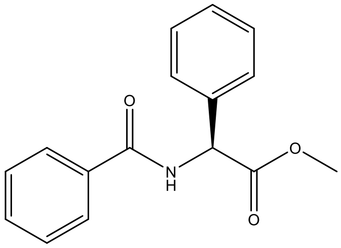

Methyl (S)-2-benzamido-2-phenylacetate was synthesized according to the general procedure outlined in the Experimental Section. The product was purified through flash chromatography (30- 35% EtOAc in i-hexane) to provide the product as a white solid in 61% isolated yield. ^{1}H NMR (400 MHz, Chloroform-*d*) δ 7.84 – 7.81 (m, 2H), 7.53 – 7.49 (m, 1H), 7.46 – 7.41 (m, 4H), 7.40 – 7.31 (m, 3H), 7.17 (d, *J* = 7.0 Hz, 1H), 5.79 (d, *J* = 7.0 Hz, 1H), 3.77 (s, 3H). ^{13}C NMR (101 MHz, Chloroform-*d*) δ 171.6, 166.5, 136.7, 133.7, 132.0, 129.2, 128.75, 128.73, 127.5, 127.3, 56.9, 53.1. HRMS: calcd. for $C_{16}H_{16}NO_3$ [M + H]+ 270.1130; found: 270.1140. $[\alpha]^{25}_{589} = +0.40$ (c = 1.01, THF).

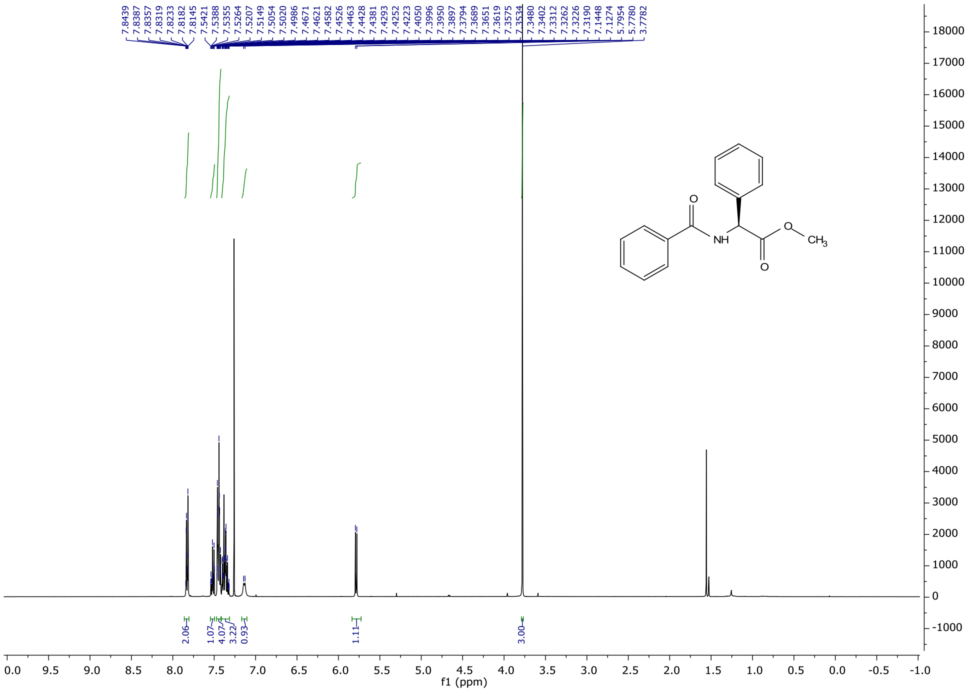

Figure S5. ^{1}H NMR (400 MHz, $CDCl_3$) spectra of methyl (S)-2-benzamido-2-phenylacetate.

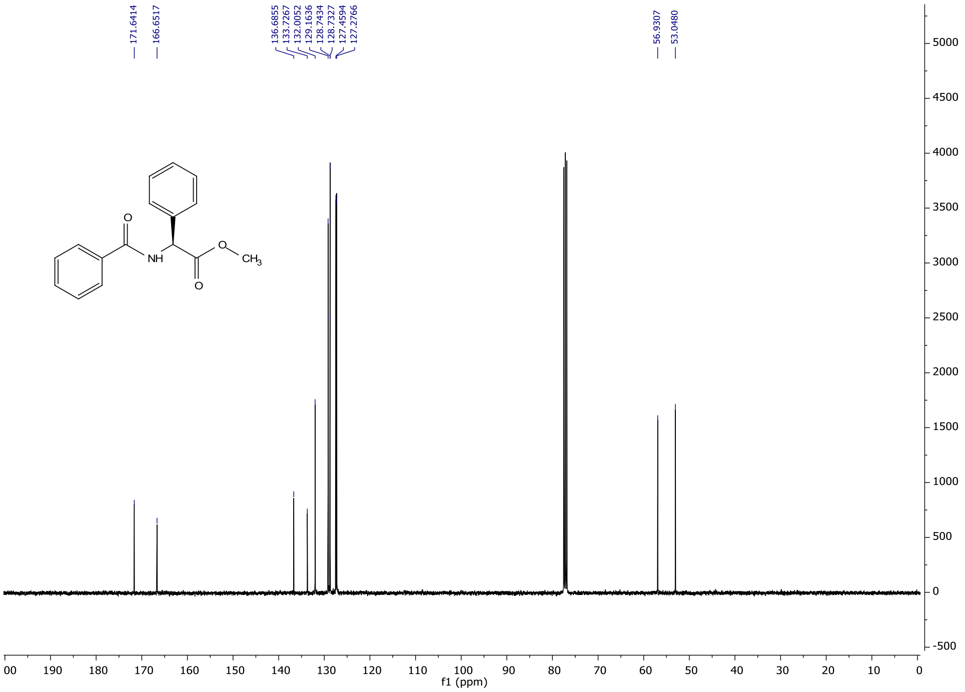

Figure S6. $^{13}C\{^{1}H\}$ NMR (101 MHz, $CDCl_3$) spectra of methyl (S)-2-benzamido-2-phenylacetate.

For methyl (S)-2-benzamido-2-phenylacetate, the same procedure outlined on page S3 was used for determination of the enantiomeric ratio with the column exchanged for a 5 µm YMC Chiral Cellulose-SB, 4.6 mm x 150 mm (diameter x length) column.

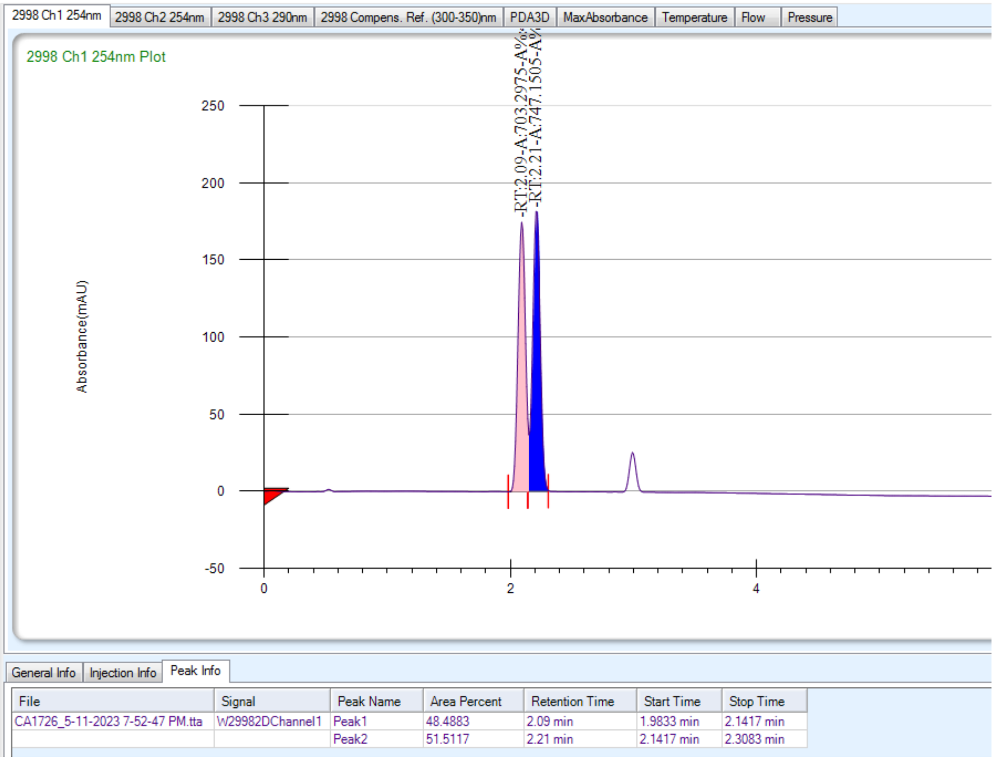

Figure S7. SFC-UV spectrum of racemic mixture of methyl 2-benzamido-2-phenylacetate.

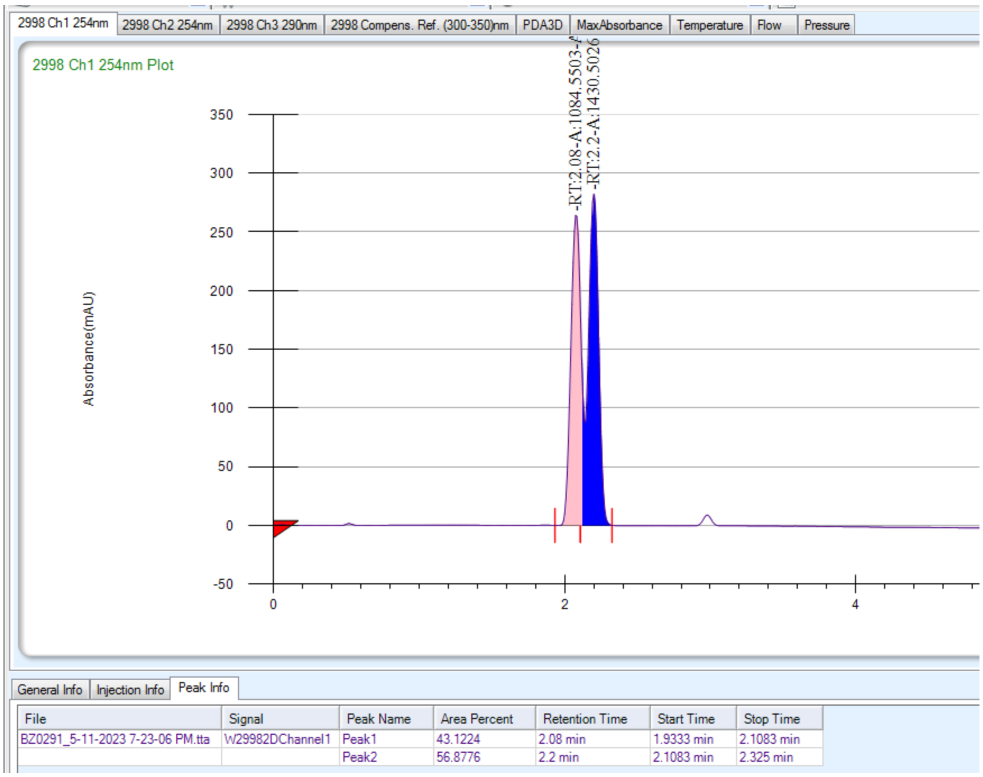

Figure S8. SFC-UV spectrum of methyl (S)-2-benzamido-2-phenylacetate synthesized through our optimized method, resulting in a racemic mixture.

NMR-spectra of compounds **3a – 4i**

***Tert*-butyl benzoyl-L-phenylalaninate (3a)**

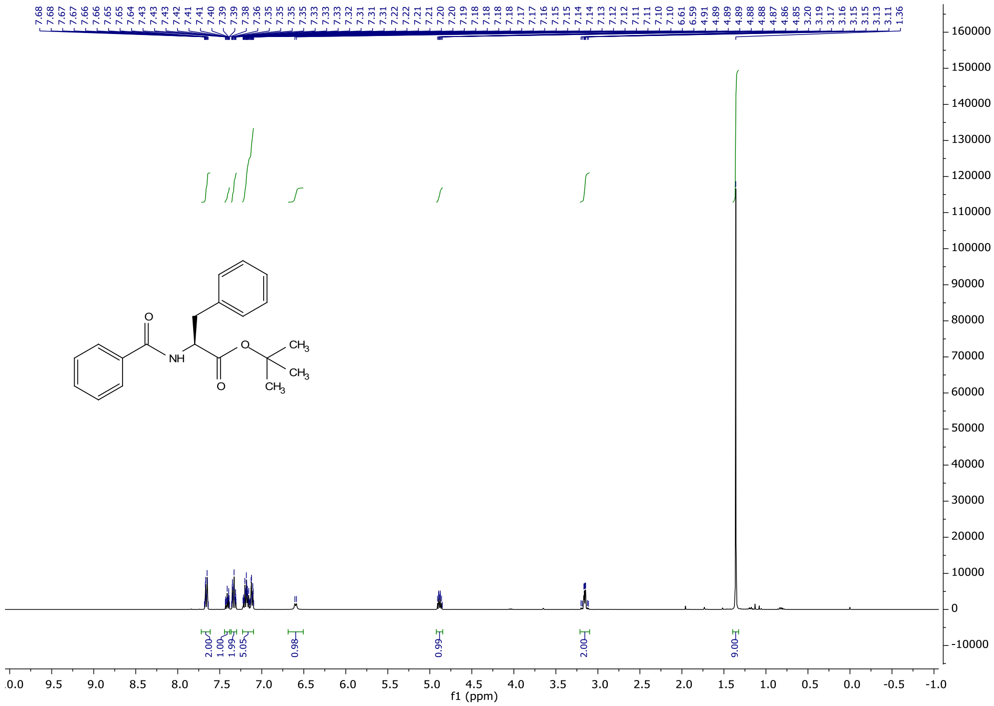

Figure S9. ^{1}H NMR (400 MHz, $CDCl_3$) spectra of **3a**.

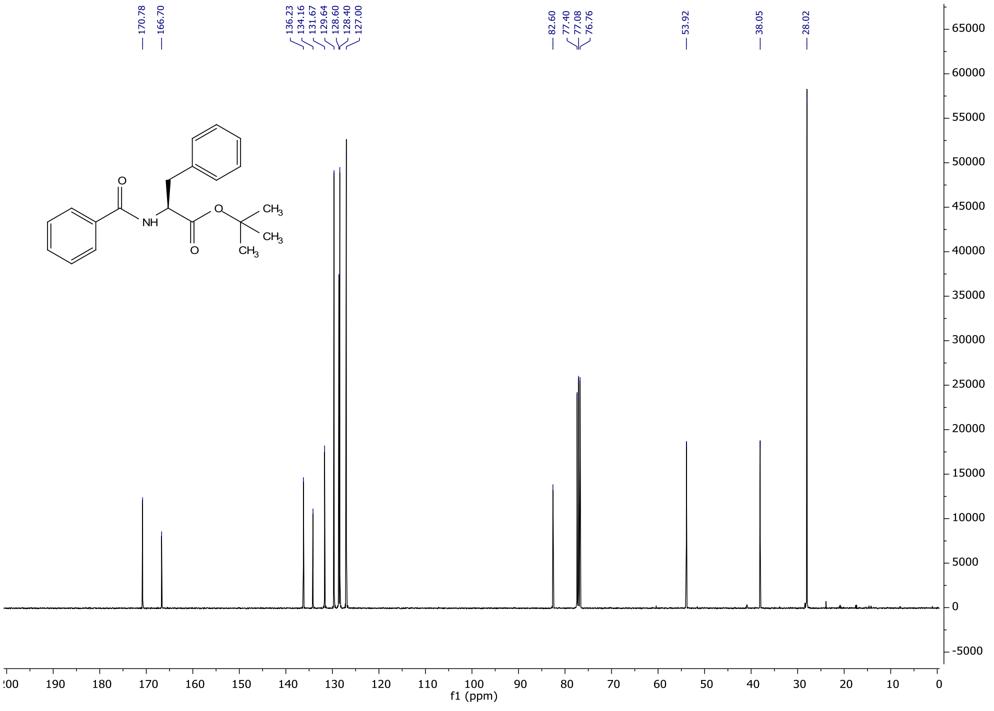

Figure S10. $^{13}C\{^{1}H\}$ NMR (101 MHz, $CDCl_3$) spectra of **3a**.

Methyl benzoyl-L-alaninate (**3b**)

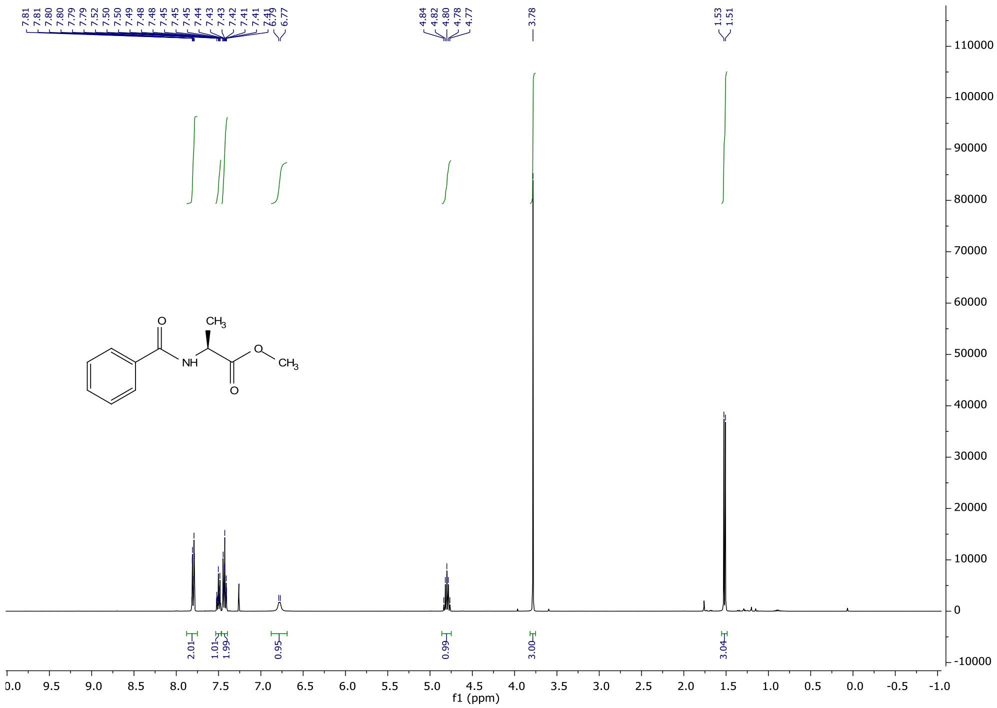

Figure S11. ^{1}H NMR (400 MHz, $CDCl_3$) spectra of **3b**.

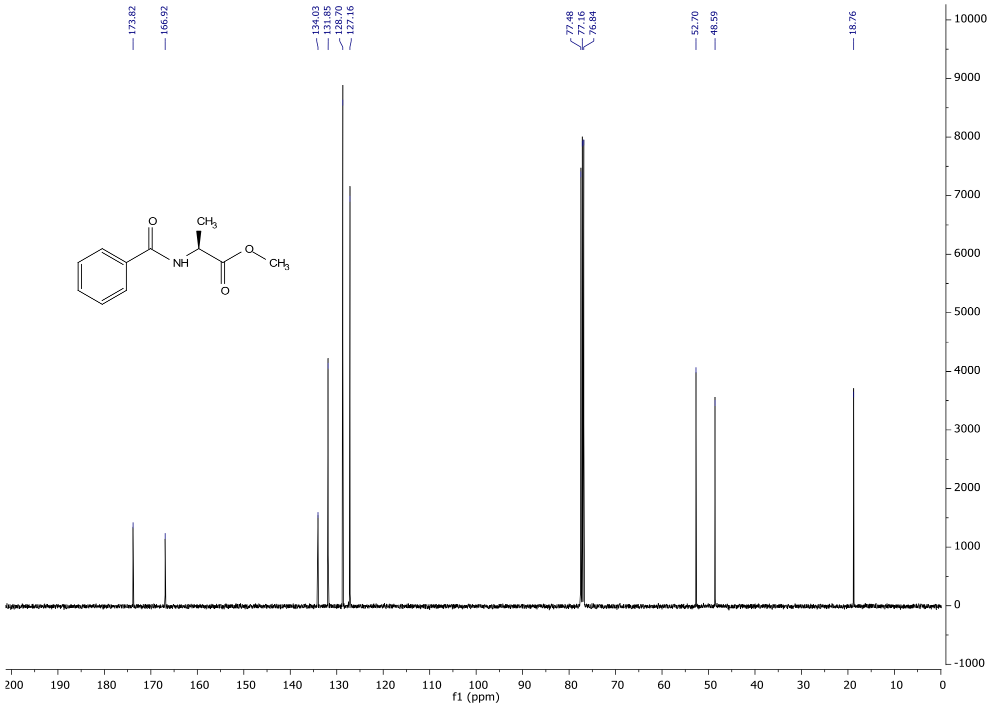

Figure S12. $^{13}C\{^{1}H\}$ NMR (101 MHz, $CDCl_3$) spectra of **3b**.

Methyl benzoyl-L-valinate (3c)

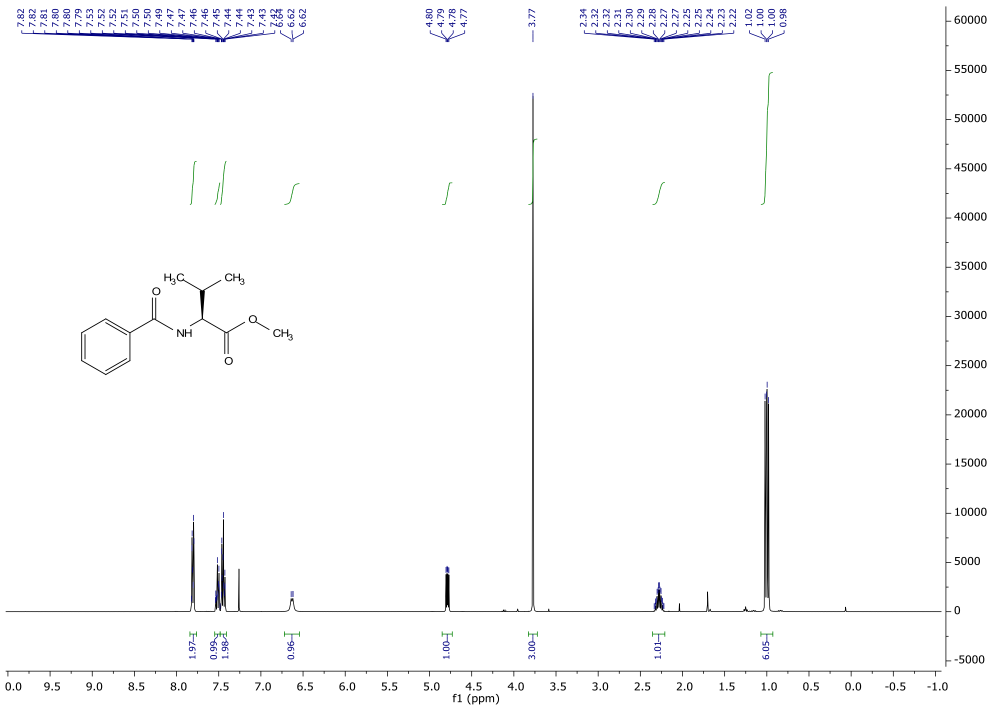

Figure S13. ^{1}H NMR (400 MHz, $CDCl_3$) spectra of **3c**.

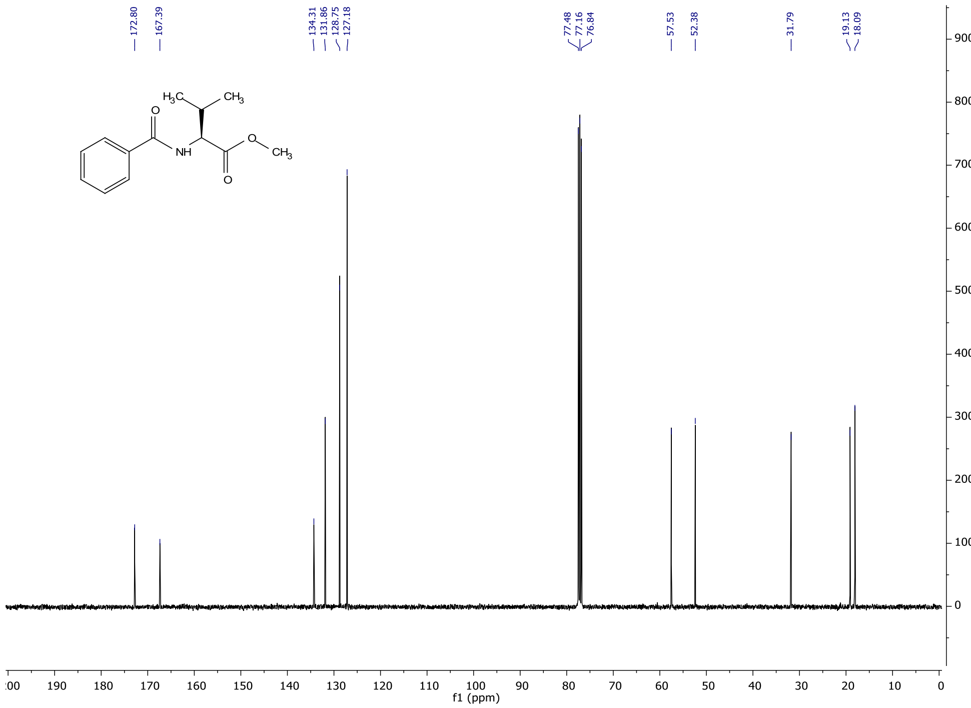

Figure S14. $^{13}C\{^{1}H\}$ NMR (101 MHz, $CDCl_3$) spectra of **3c**.

Methyl benzoyl-L-isoleucinate (3d)

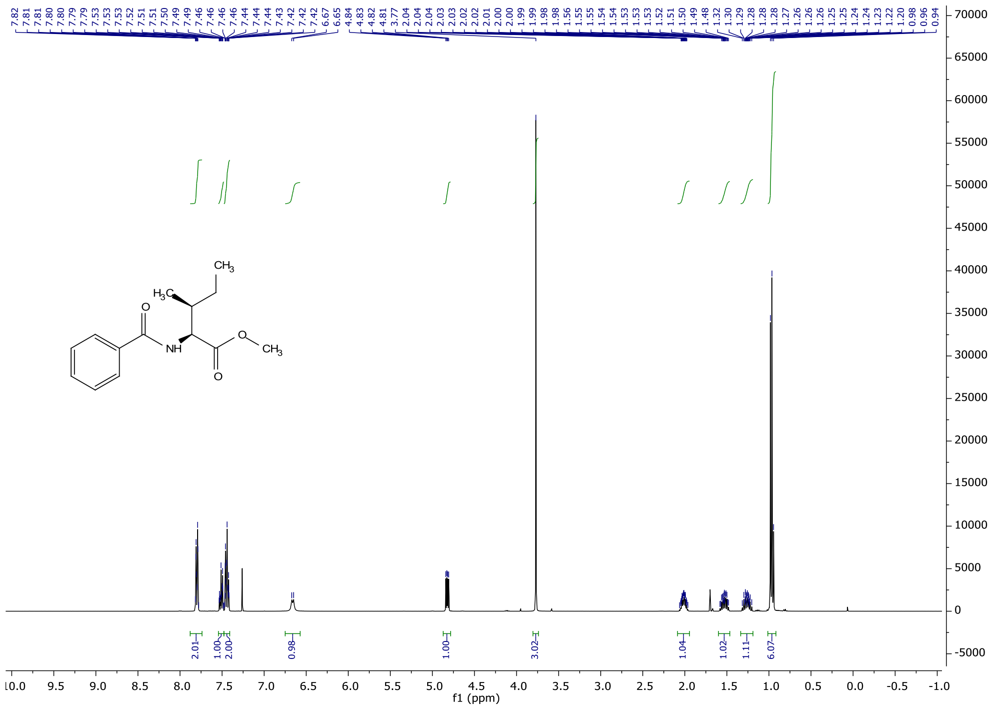

Figure S15. ^{1}H NMR (400 MHz, $CDCl_3$) spectra of **3d**.

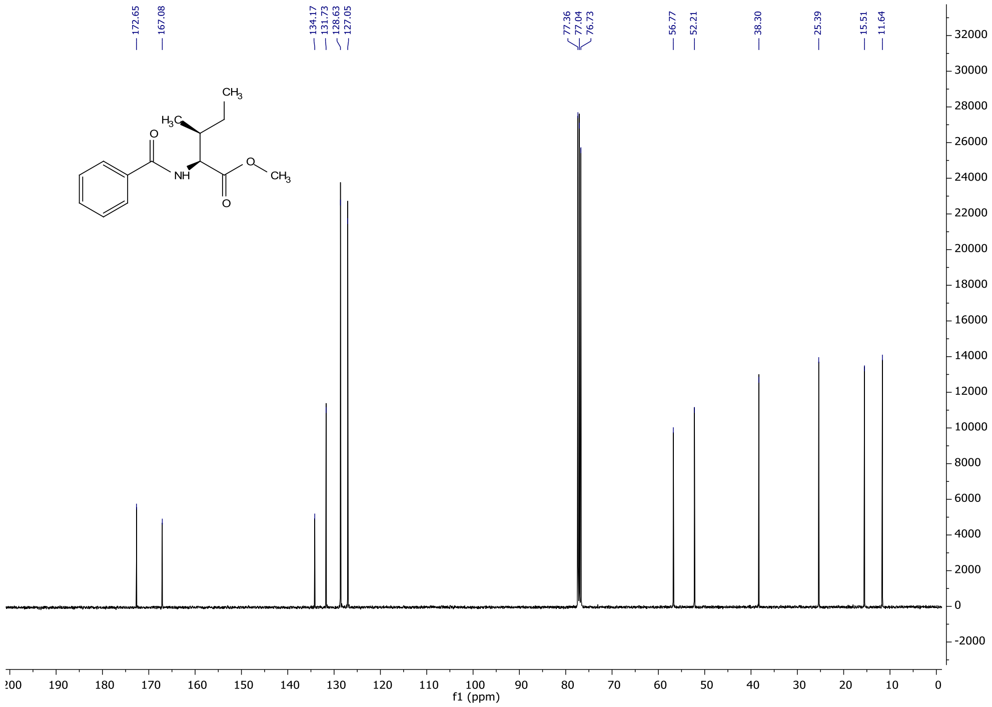

Figure S16. $^{13}C\{^1H\}$ NMR (101 MHz, $CDCl_3$) spectra of **3d**.

Methyl benzoyl-L-leucinate (3e)

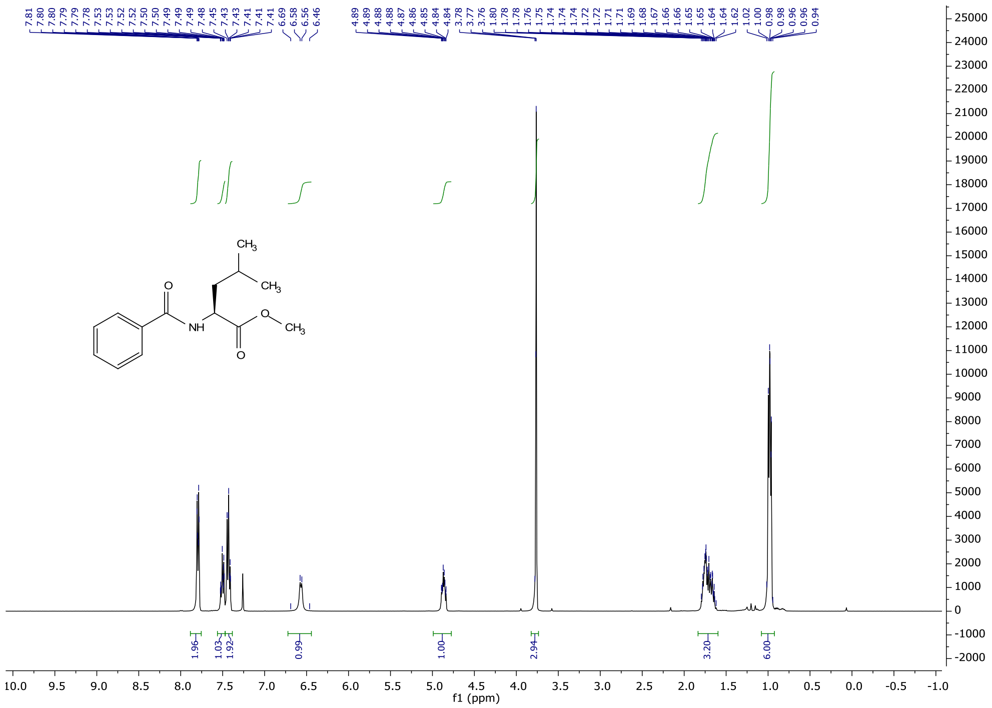

Figure S17. ^{1}H NMR (400 MHz, $CDCl_3$) spectra of **3e**.

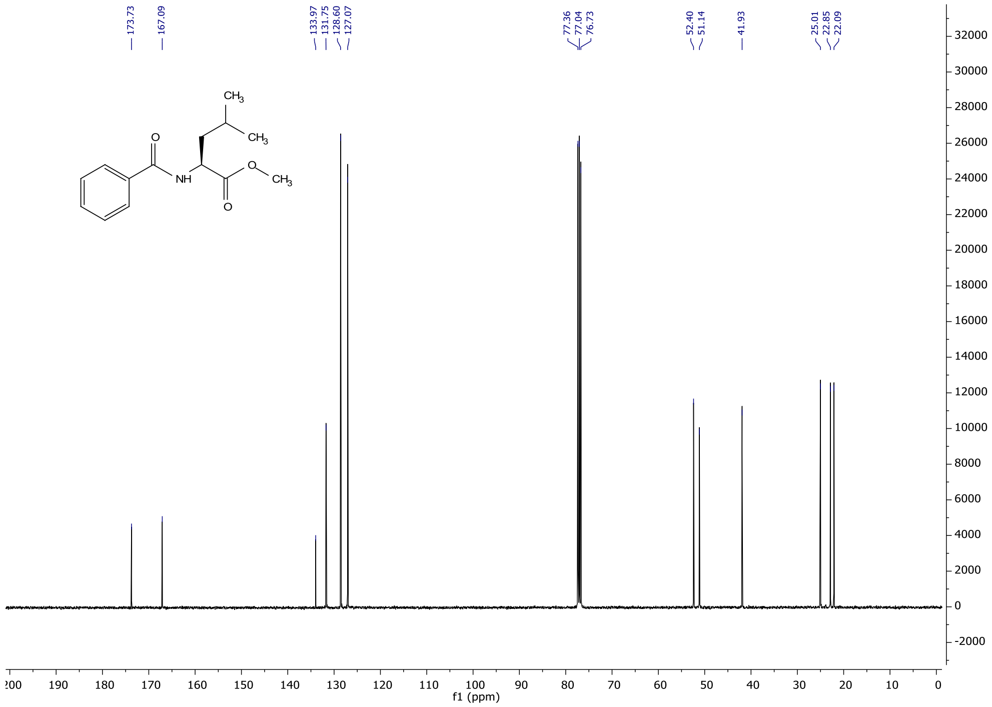

Figure S18. $^{13}C\{^{1}H\}$ NMR (101 MHz, $CDCl_3$) spectra of **3e**.

Ethyl (2*R*)-2-benzamido-2-phenylacetate (3f)

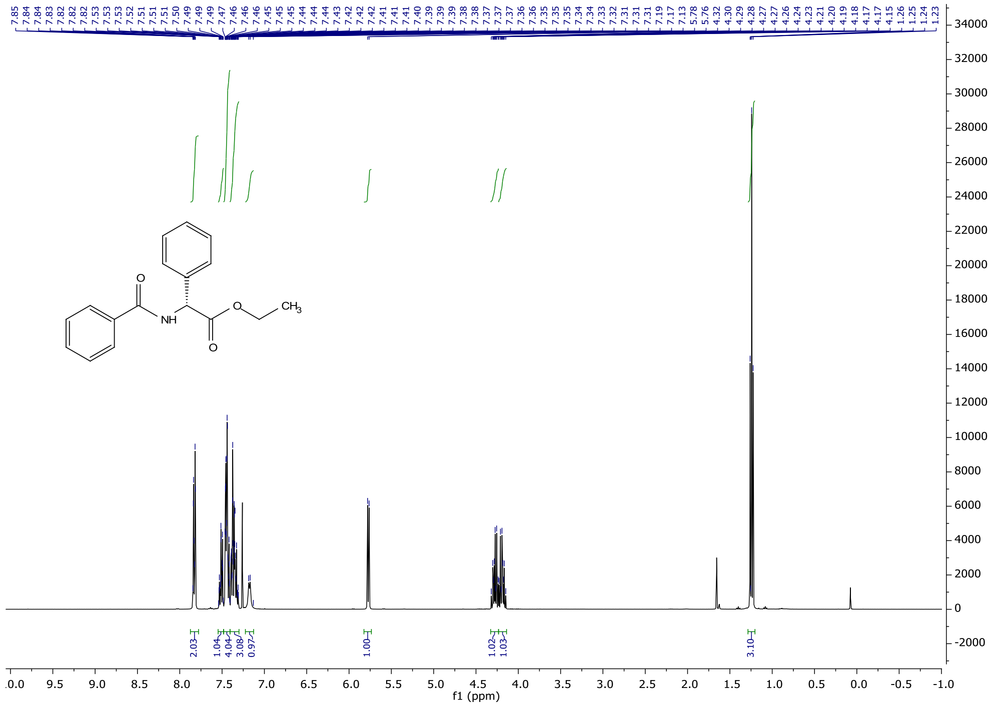

Figure S19. ^{1}H NMR (400 MHz, $CDCl_3$) spectra of **3f**.

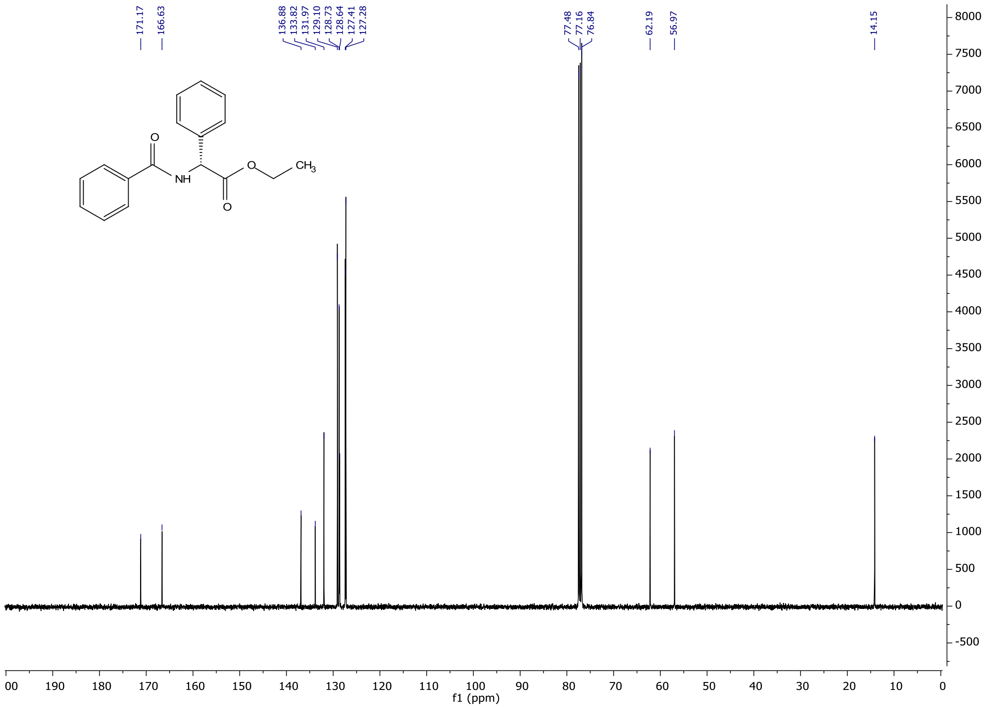

Figure S20. $^{13}C\{^{1}H\}$ NMR (101 MHz, $CDCl_3$) spectra of **3f**.

Methyl (*S*)-2-benzamido-3-(4-*tert*-butoxyphenyl)propanoate (3g)

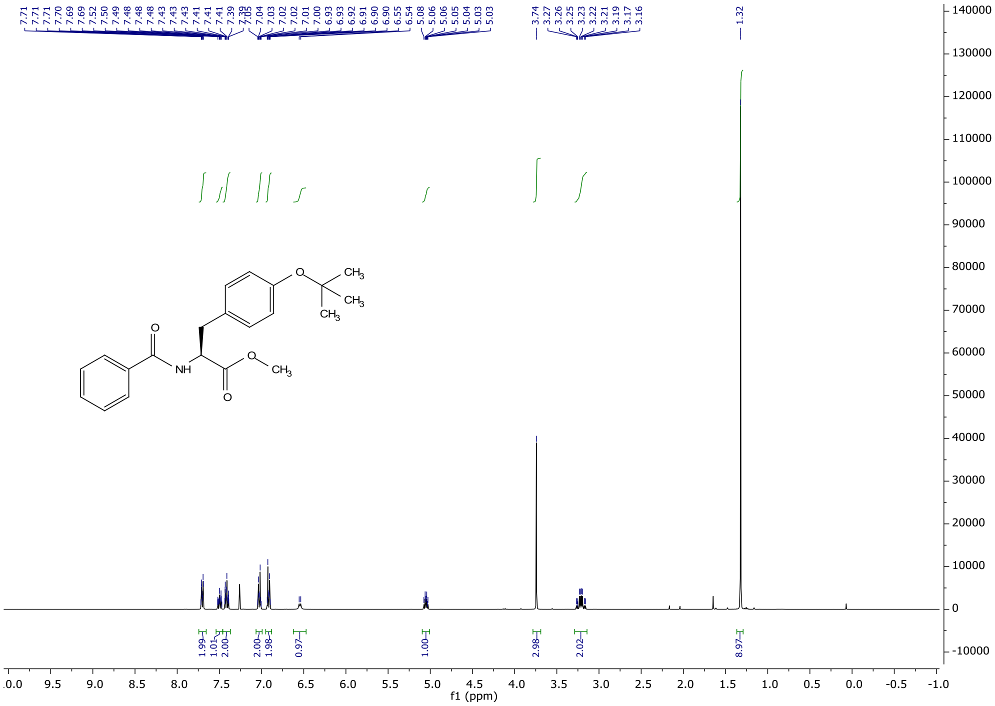

Figure S21. ^{1}H NMR (400 MHz, $CDCl_3$) spectra of **3g**.

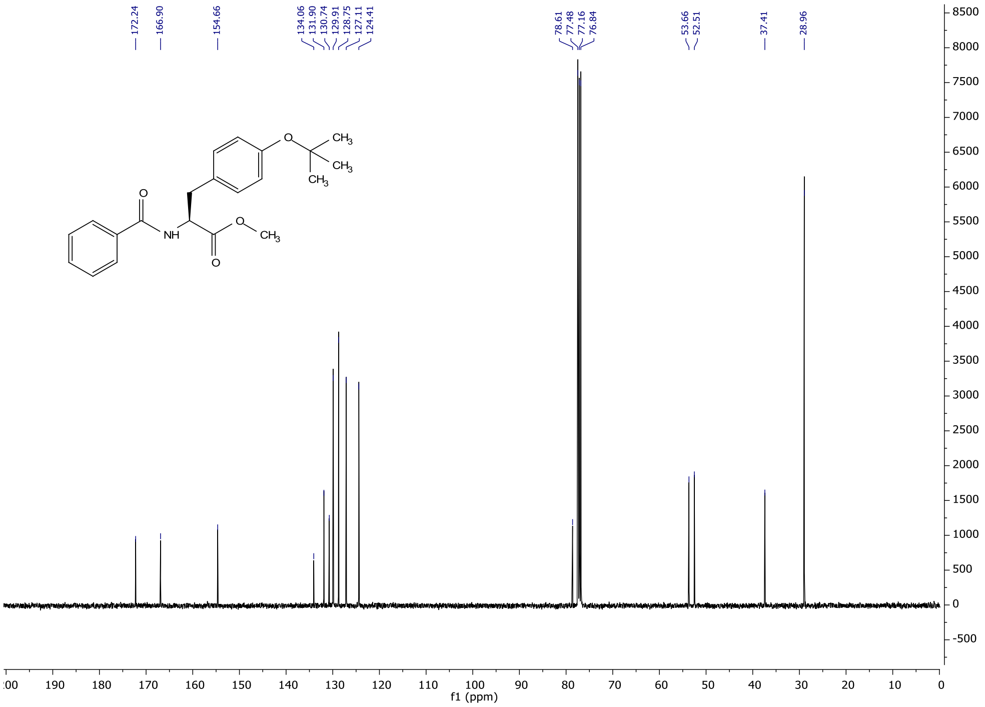

Figure S22. $^{13}C\{^{1}H\}$ NMR (101 MHz, $CDCl_3$) spectra of **3g**.

Methyl *N*-benzoyl-*O*-(*tert*-butyl)-L-serinate (3h)

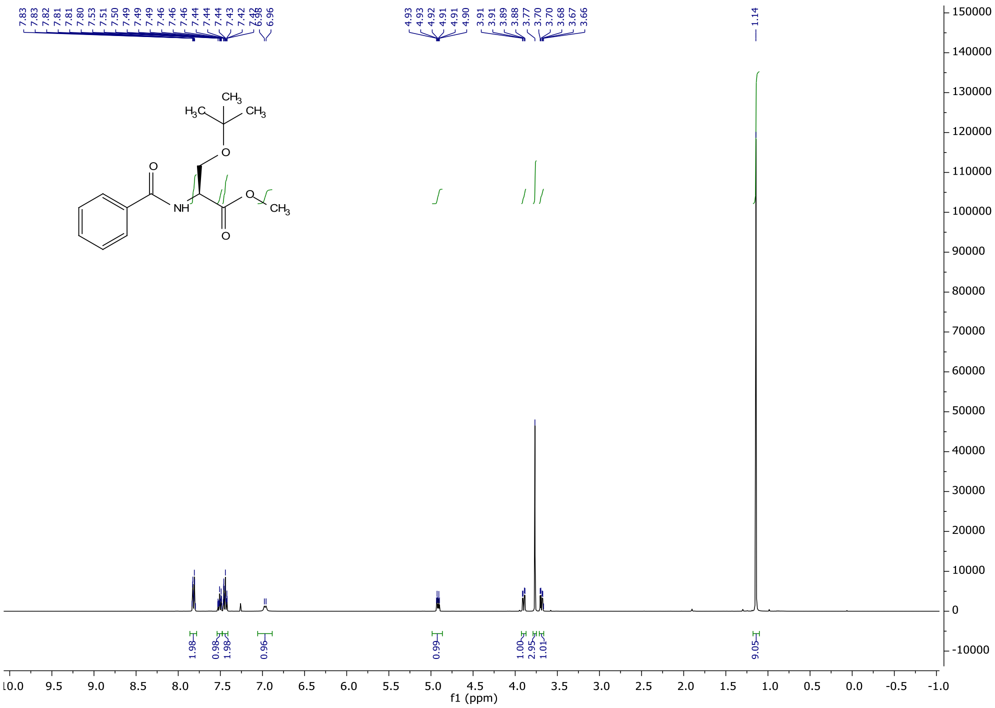

Figure S23. ^{1}H NMR (400 MHz, $CDCl_3$) spectra of **3h**.

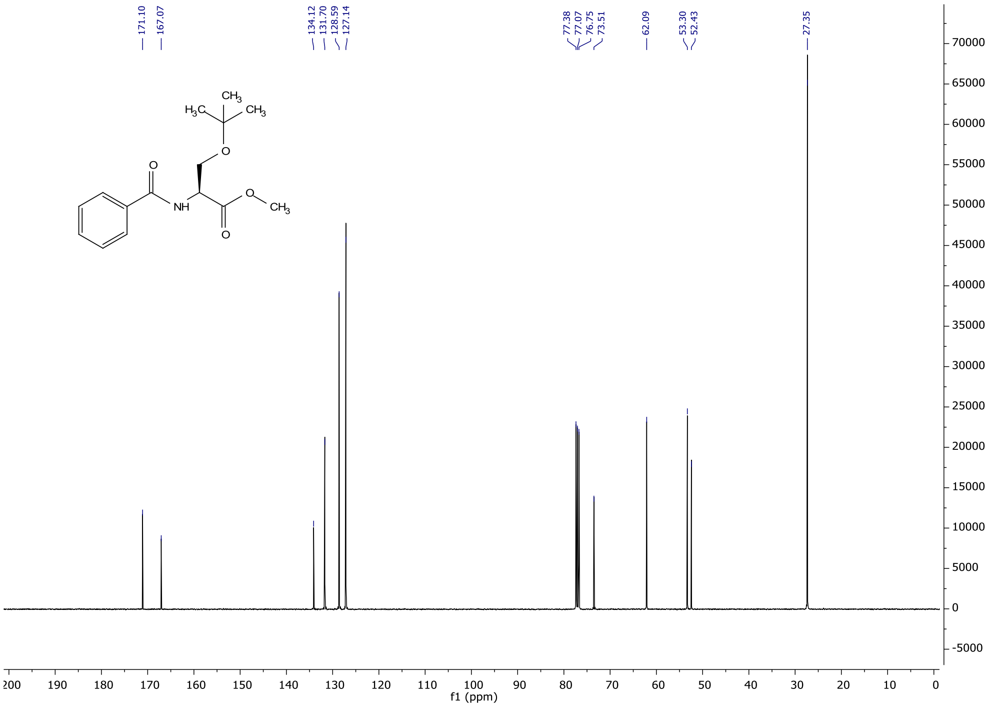

Figure S24. $^{13}C\{^{1}H\}$ NMR (101 MHz, $CDCl_3$) spectra of **3h**.

Methyl *N*-benzoyl-*O*-(*tert*-butyl)-L-threoninate (3i)

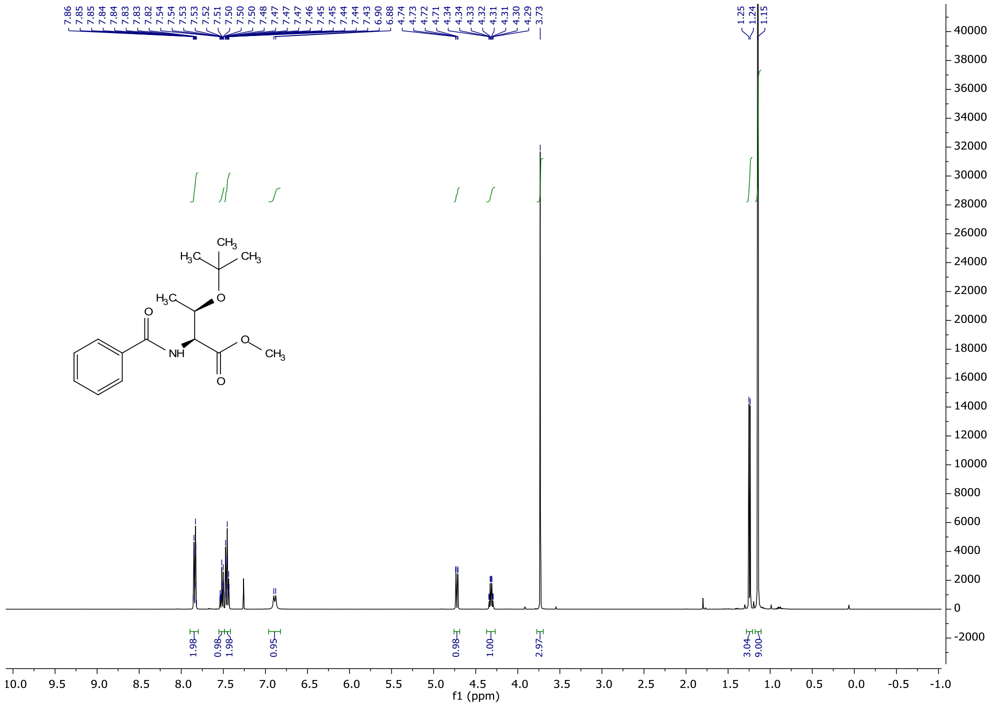

Figure S25. ^{1}H NMR (400 MHz, $CDCl_3$) spectra of **3i**.

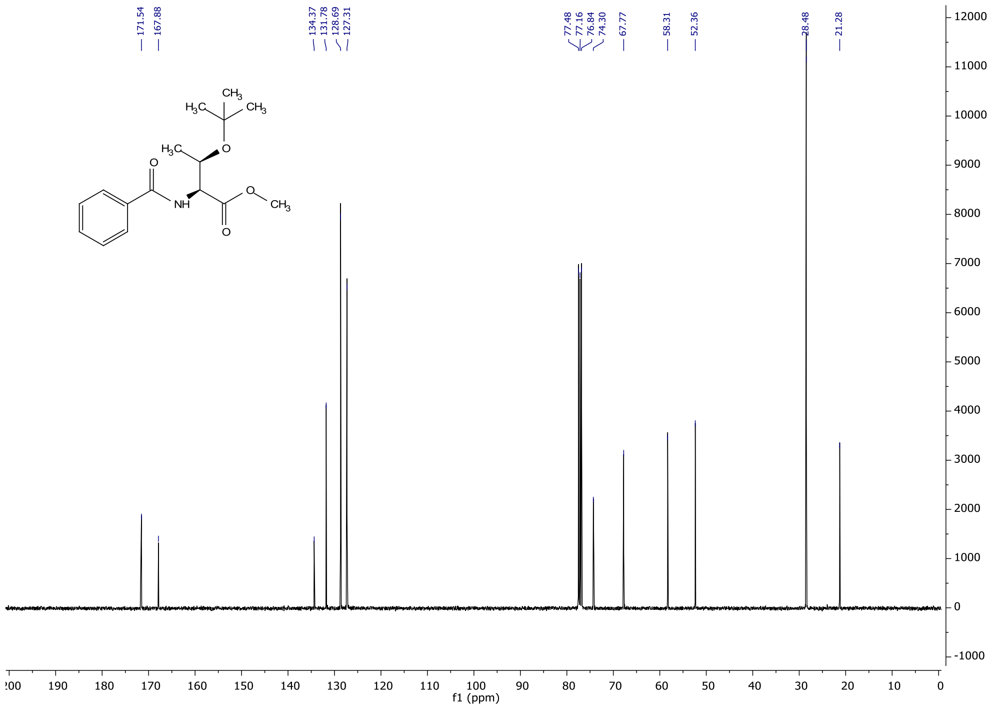

Figure S26. $^{13}C\{^1H\}$ NMR (101 MHz, $CDCl_3$) spectra of **3i**.

5-(*tert*-butyl) 1-methyl benzoyl-L-glutamate (3j)

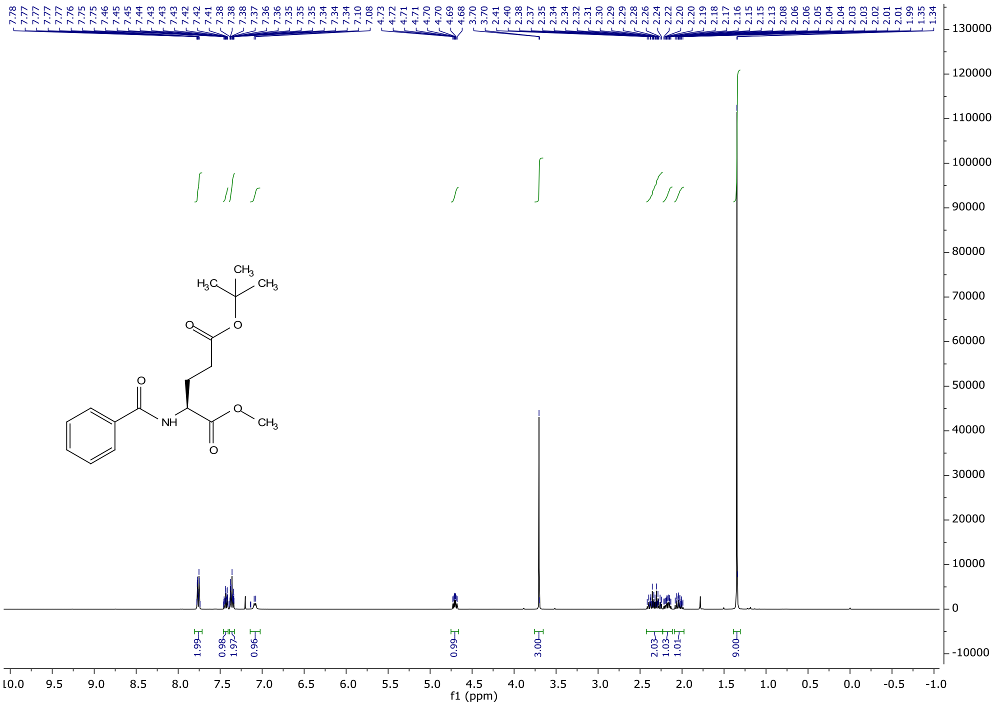

Figure S27. ^{1}H NMR (400 MHz, $CDCl_3$) spectra of **3j**.

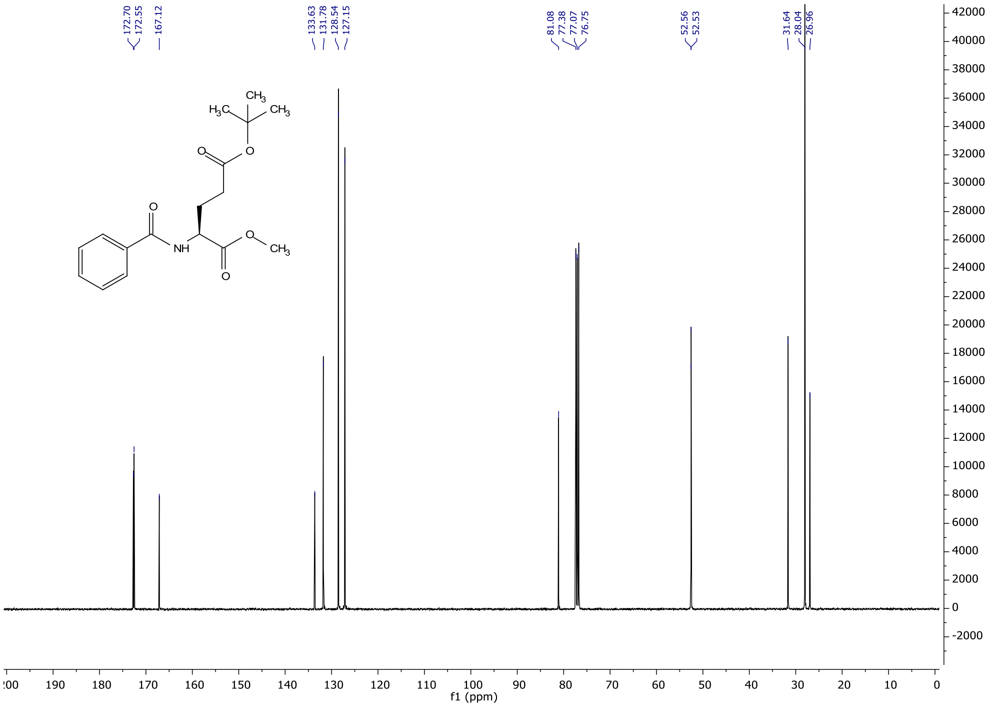

Figure S28. $^{13}C\{^{1}H\}$ NMR (101 MHz, $CDCl_3$) spectra of **3j**.

***Tert*-butyl benzoyl-L-prolinate (3k)**

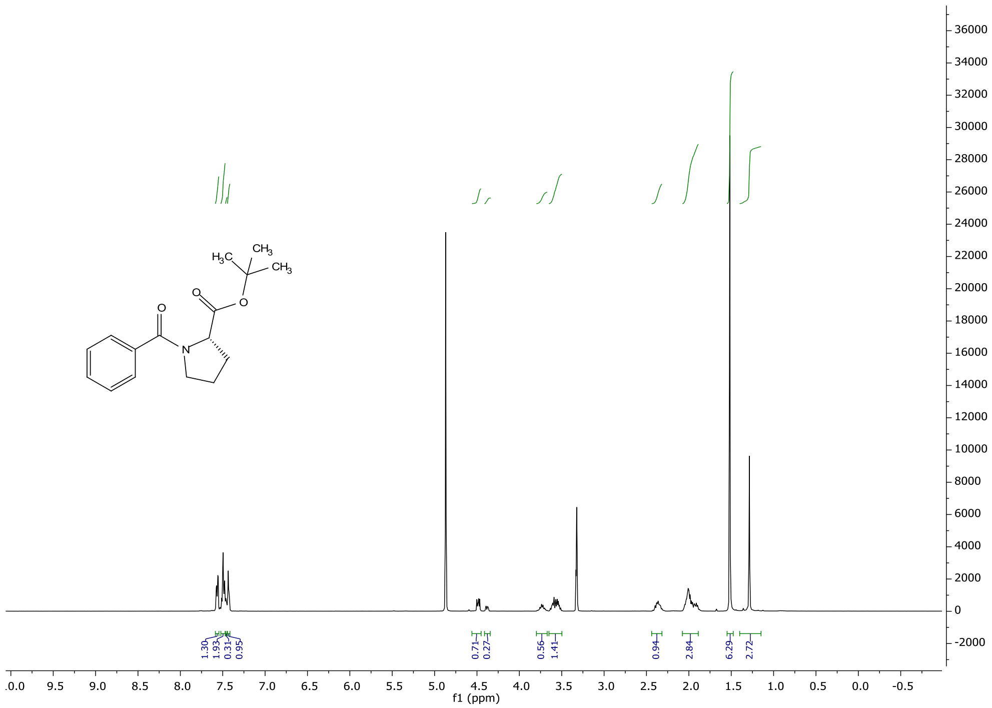

Figure S29. ^{1}H NMR (400 MHz, MeOD-d4) spectra of **3k**.

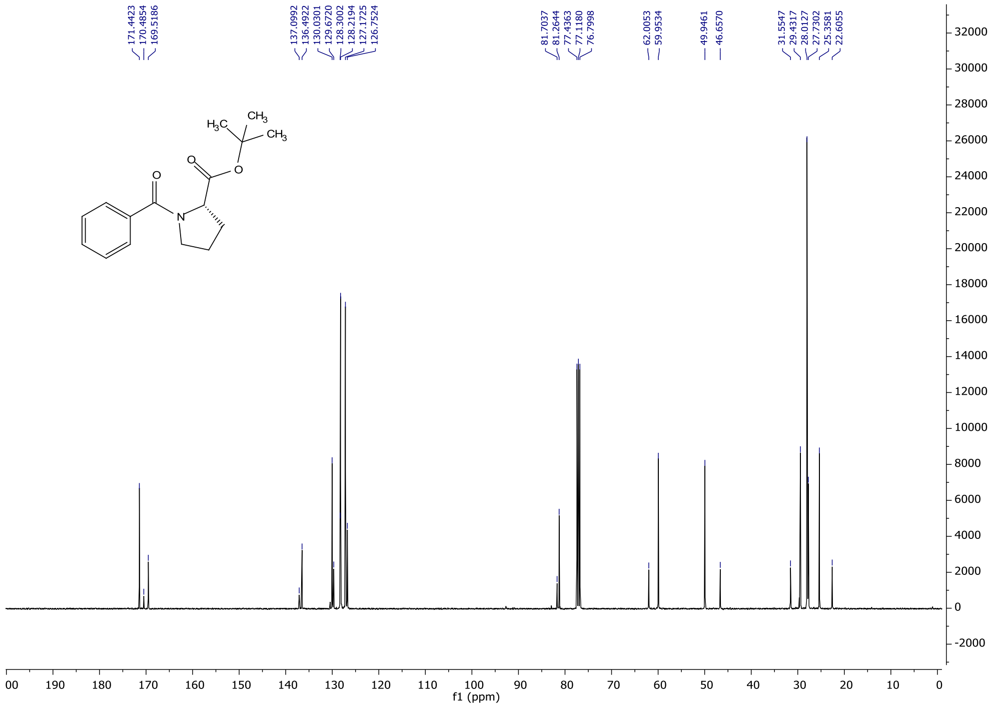

Figure S30. $^{13}C\{^{1}H\}$ NMR (101 MHz, $CDCl_3$) spectra of **3k**.

Methyl N^2-benzoyl-N^6-(*tert*-butoxycarbonyl)-L-lysinate (**3l**)

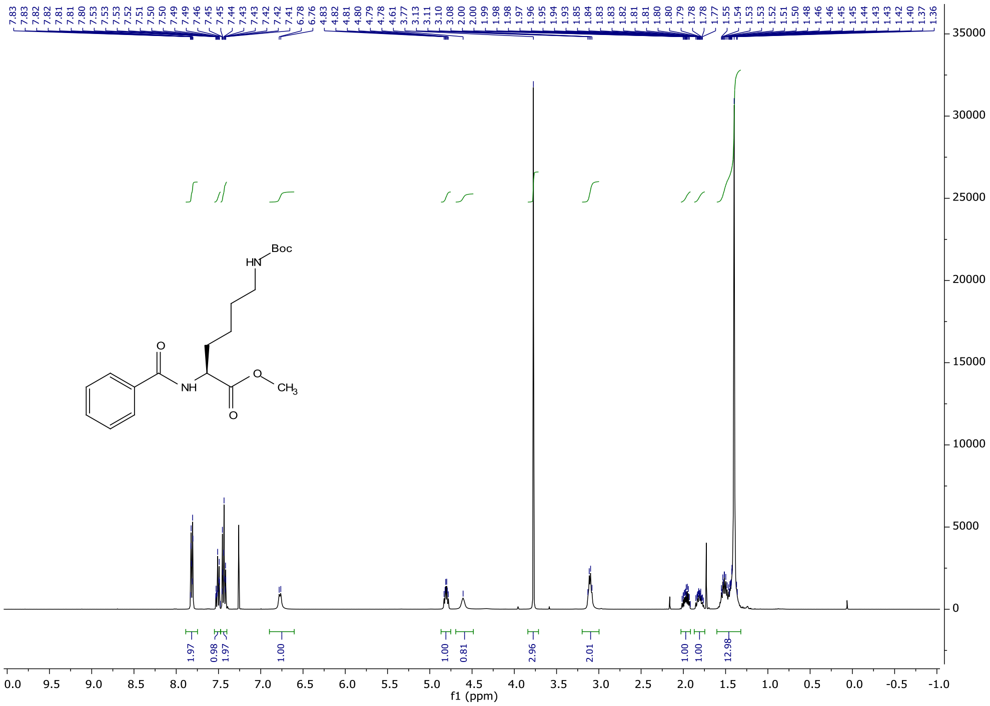

Figure S31. ^{1}H NMR (400 MHz, $CDCl_3$) spectra of **3l**.

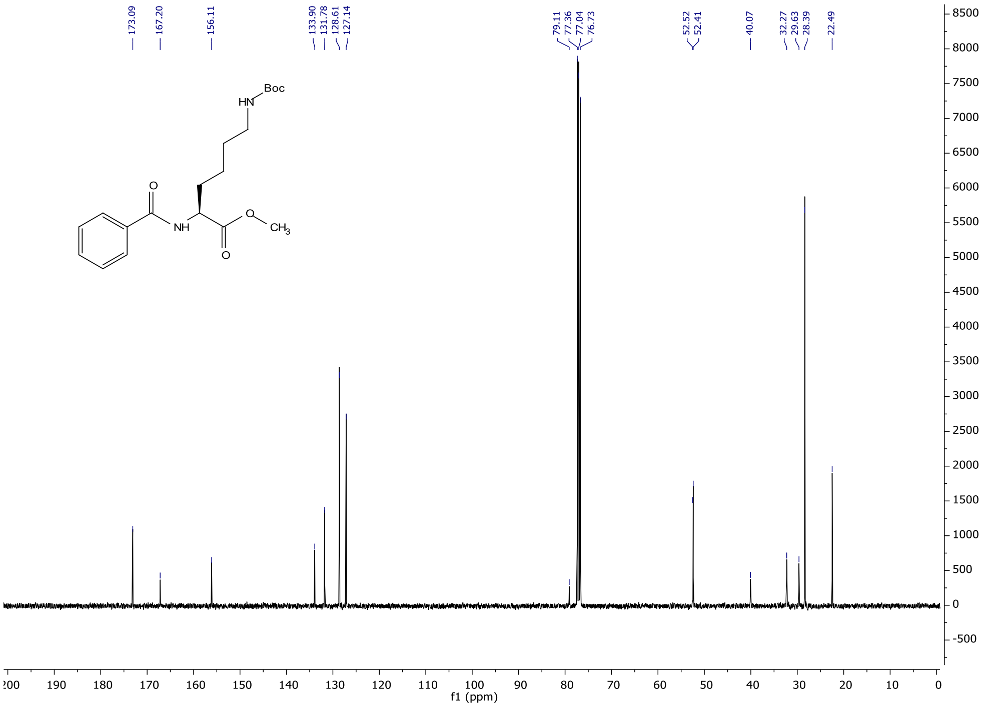

Figure S32. $^{13}C\{^1H\}$ NMR (101 MHz, $CDCl_3$) spectra of **3l**.

Methyl (4-methylbenzoyl)-L-phenylalaninate (4a)

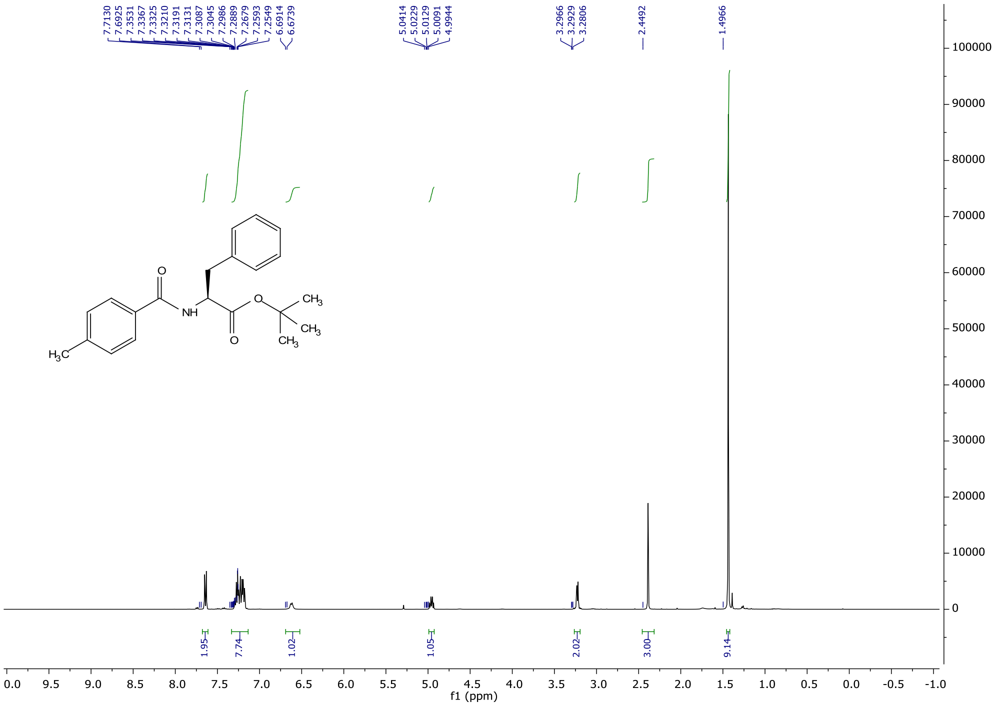

Figure S33. ^{1}H NMR (400 MHz, $CDCl_3$) spectra of **4a**.

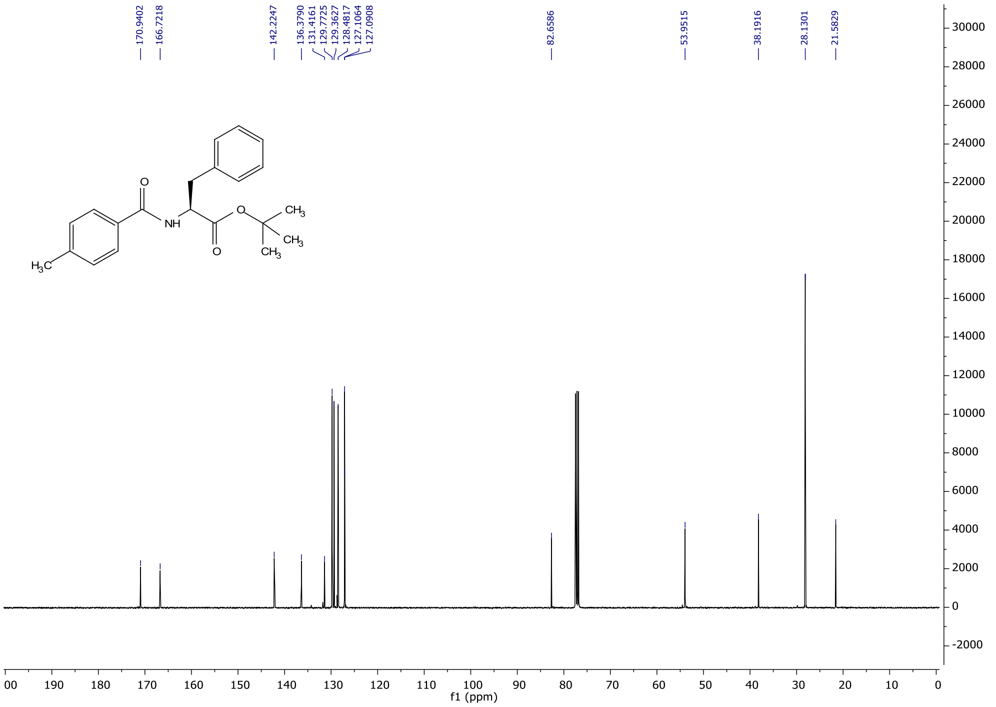

Figure S34. $^{13}C\{^{1}H\}$ NMR (101 MHz, $CDCl_3$) spectra of **4a**.

Methyl (2-methylbenzoyl)-L-phenylalaninate (4b)

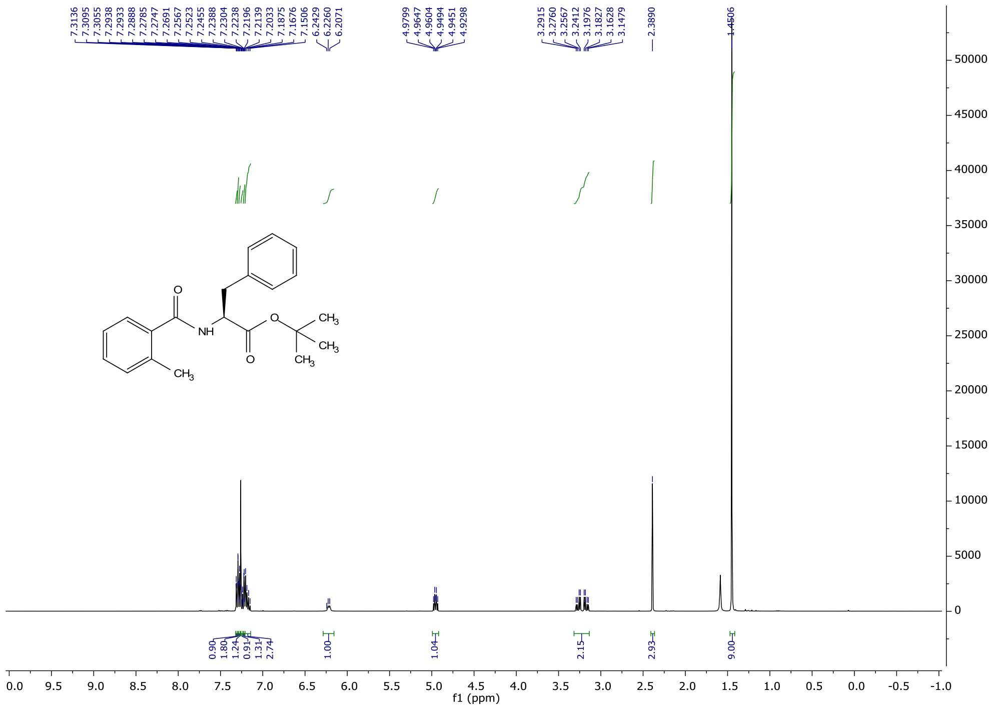

Figure S35. ^1H NMR (400 MHz, $CDCl_3$) spectra of **4b**.

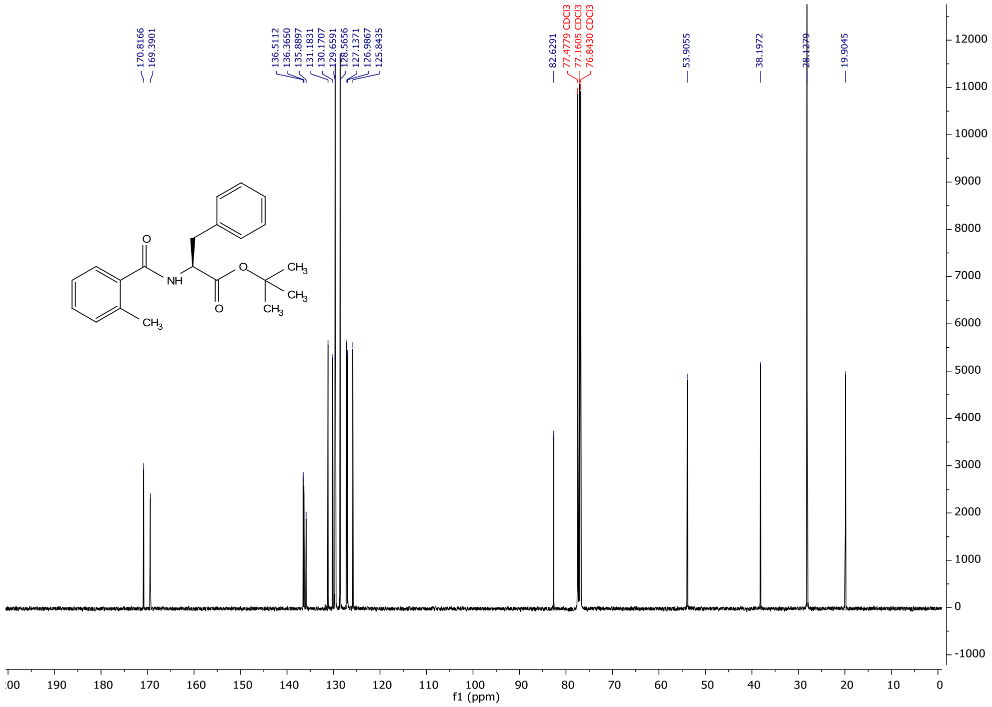

Figure S36. $^{13}C\{^1H\}$ NMR (101 MHz, $CDCl_3$) spectra of **4b**.

Methyl (2-naphthoyl)-L-phenylalaninate (4c)

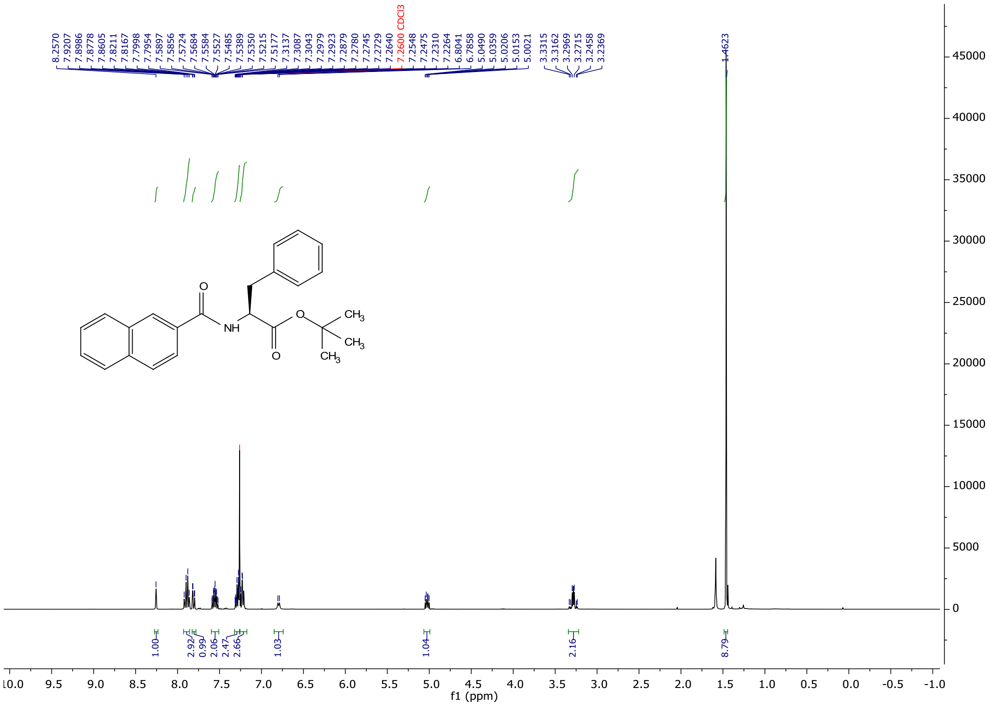

Figure S37. ^{1}H NMR (400 MHz, $CDCl_3$) spectra of **4c**.

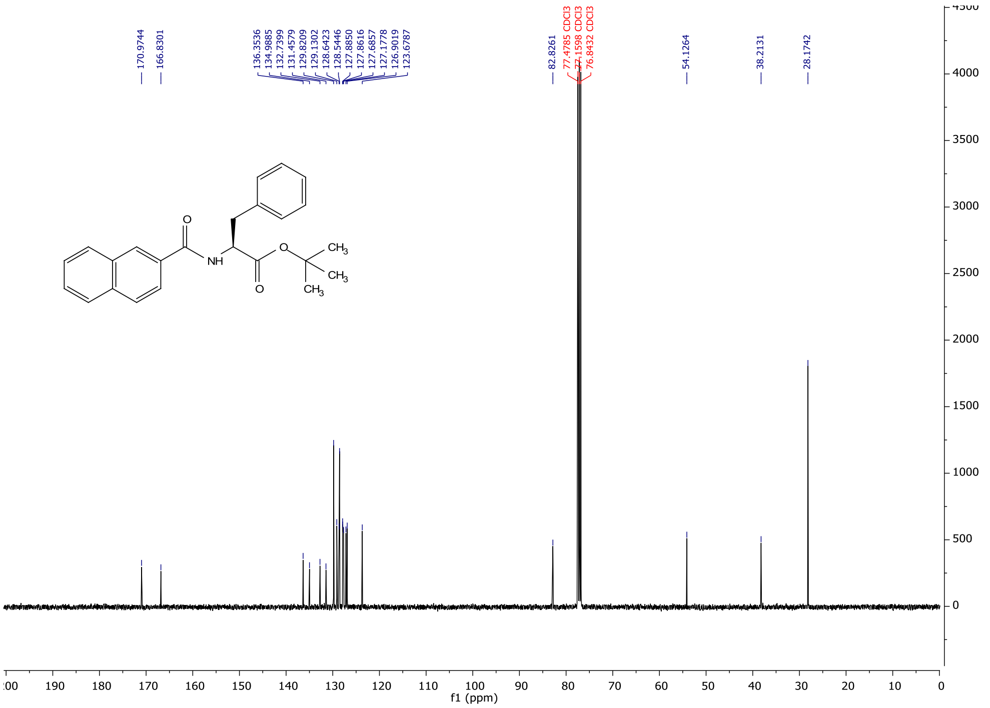

Figure S38. $^{13}C\{^{1}H\}$ NMR (101 MHz, $CDCl_3$) spectra of **4c**.

***Tert*-butyl (4-methoxybenzoyl)-L-phenylalaninate (4d)**

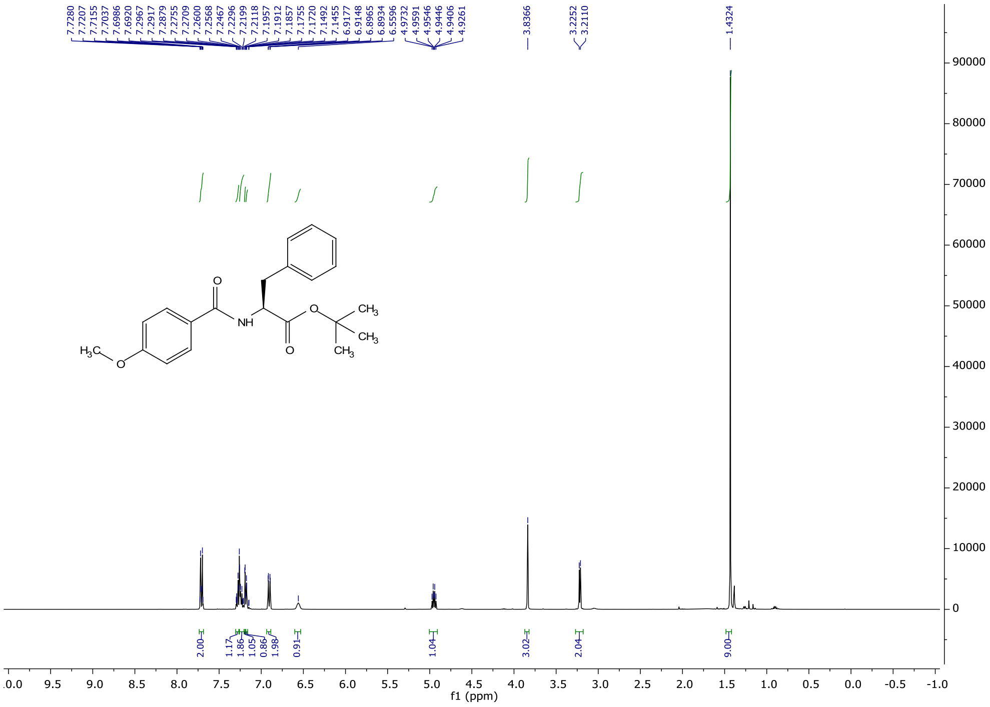

Figure S39. ^1H NMR (400 MHz, $CDCl_3$) spectra of **4d**.

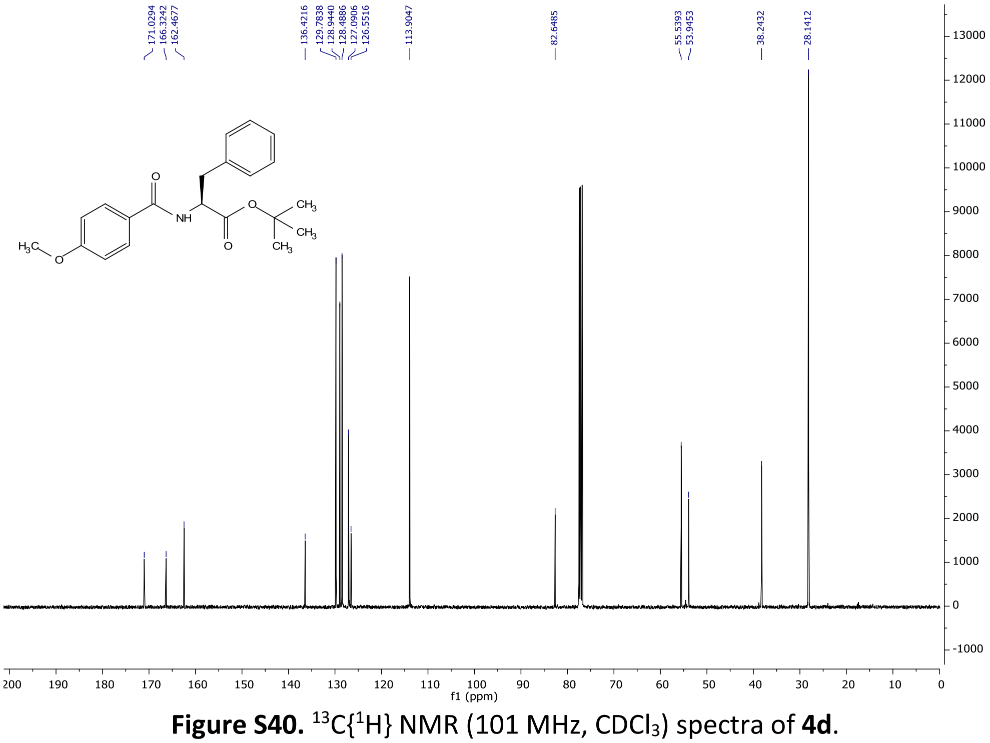

Figure S40. $^{13}C\{^1H\}$ NMR (101 MHz, $CDCl_3$) spectra of **4d**.

***Tert*-butyl (4-cyanobenzoyl)-L-phenylalaninate (4e)**

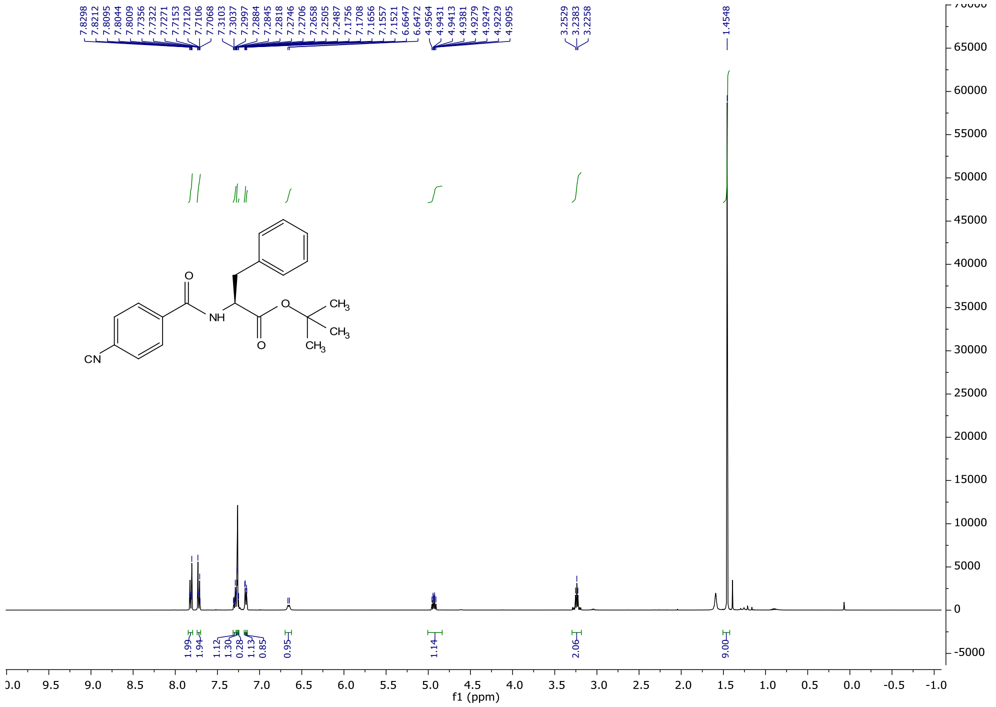

Figure S41. ^{1}H NMR (400 MHz, $CDCl_3$) spectra of **4e**.

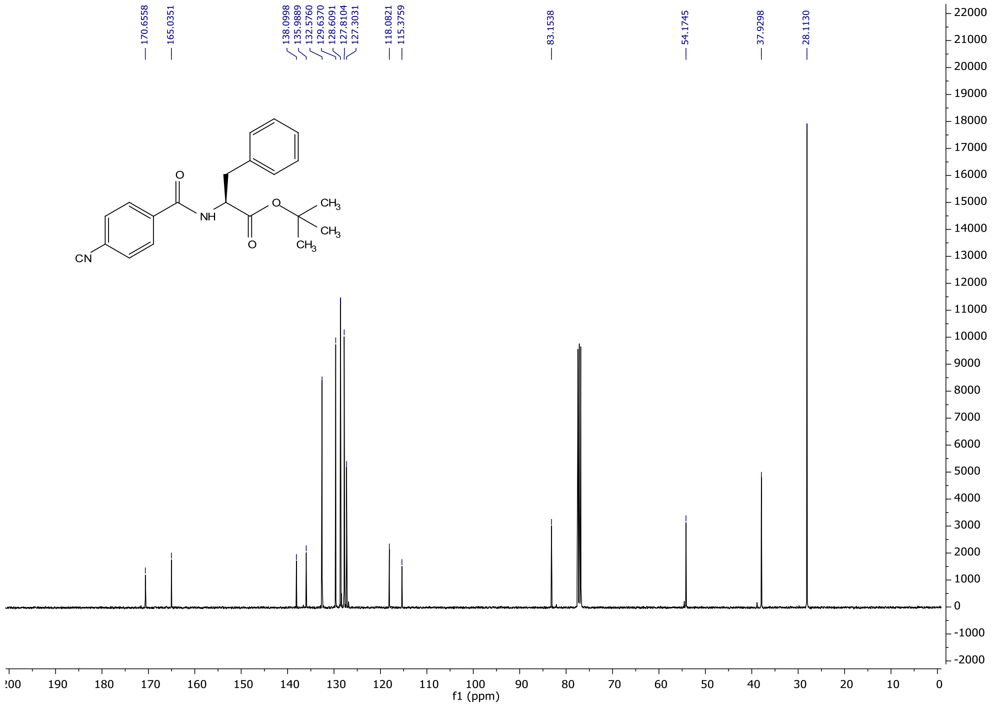

Figure S42. $^{13}C\{^{1}H\}$ NMR (101 MHz, $CDCl_3$) spectra of **4e**.

Tert-butyl nicotinoyl-L-phenylalaninate (4f)

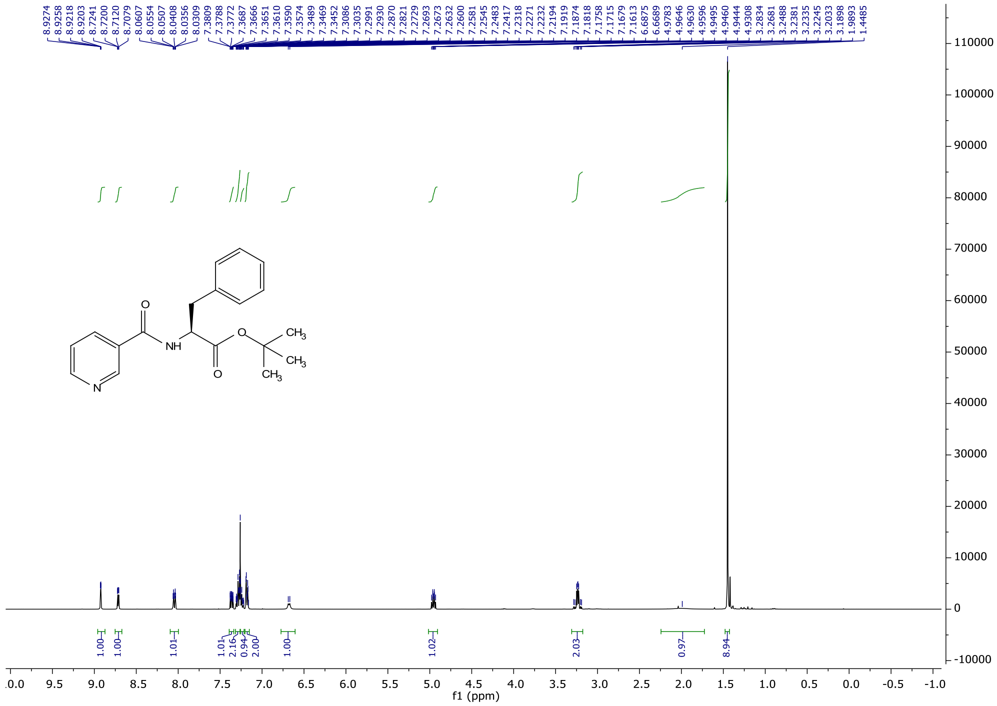

Figure S43. ^{1}H NMR (400 MHz, CDCl$_3$) spectra of **4f**.

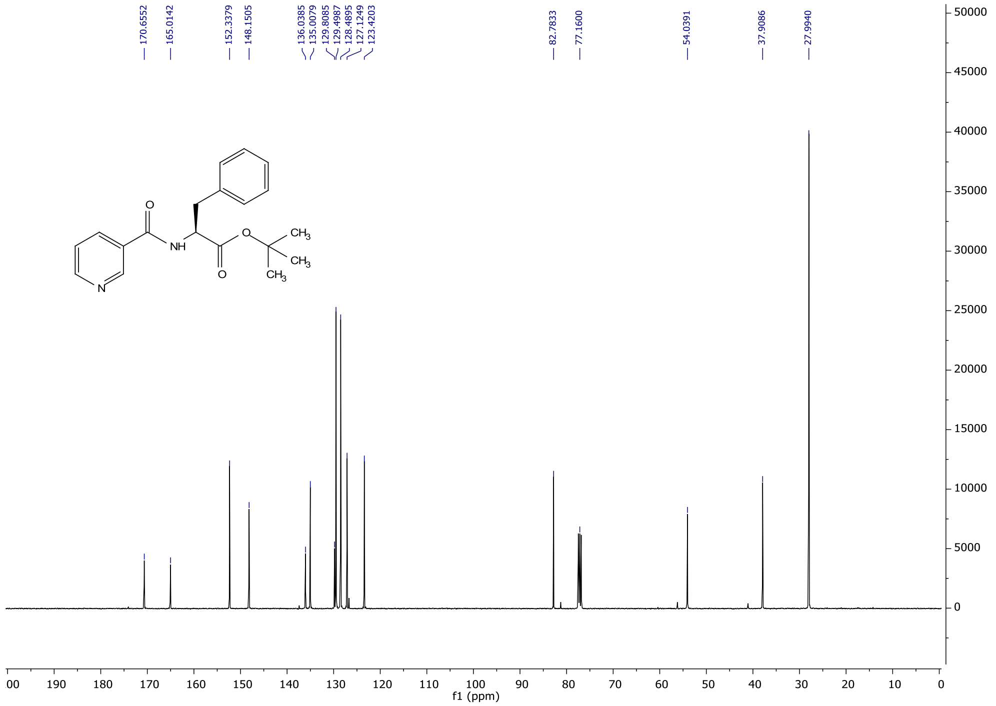

Figure S44. ^{13}C{^{1}H} NMR (101 MHz, CDCl$_3$) spectra of **4f**.

***Tert*-butyl (1H-indole-5-carbonyl)-L-phenylalaninate (4g)**

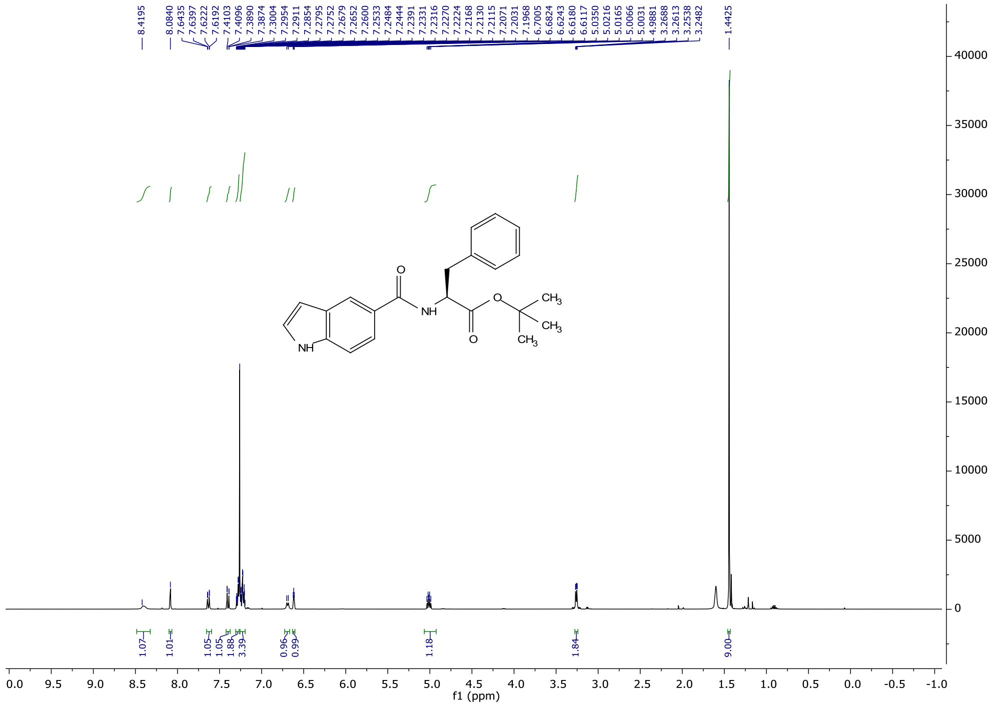

Figure S45. ^{1}H NMR (400 MHz, $CDCl_3$) spectra of **4g**.

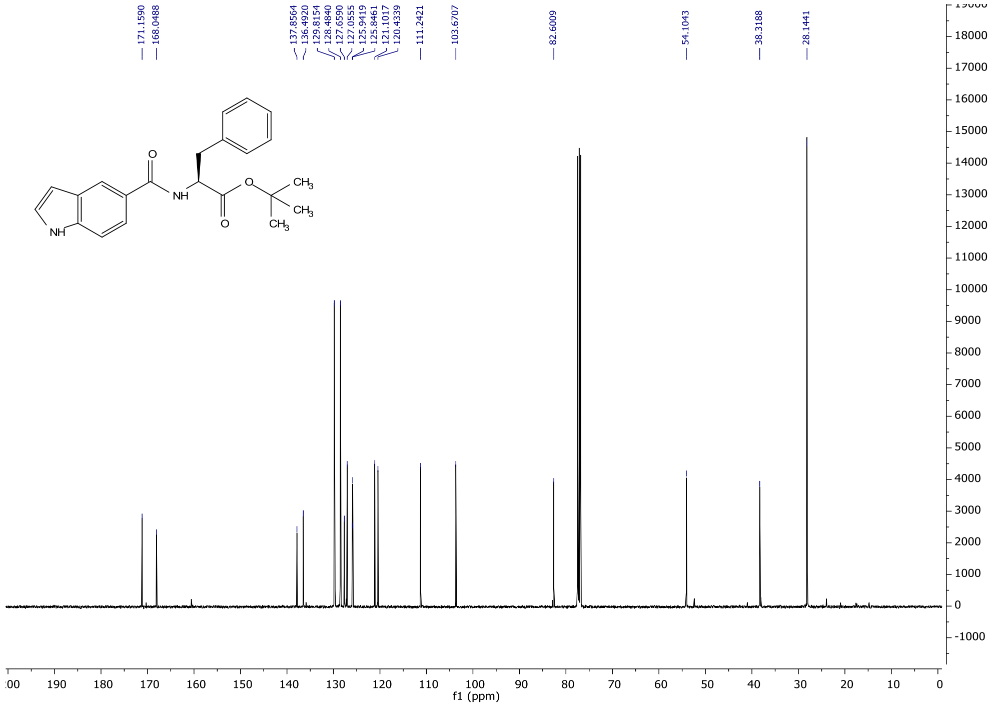

Figure S46. $^{13}C\{^{1}H\}$ NMR (101 MHz, $CDCl_3$) spectra of **4g**.

***Tert*-butyl (thiophene-2-carbonyl)-L-phenylalaninate (4h)**

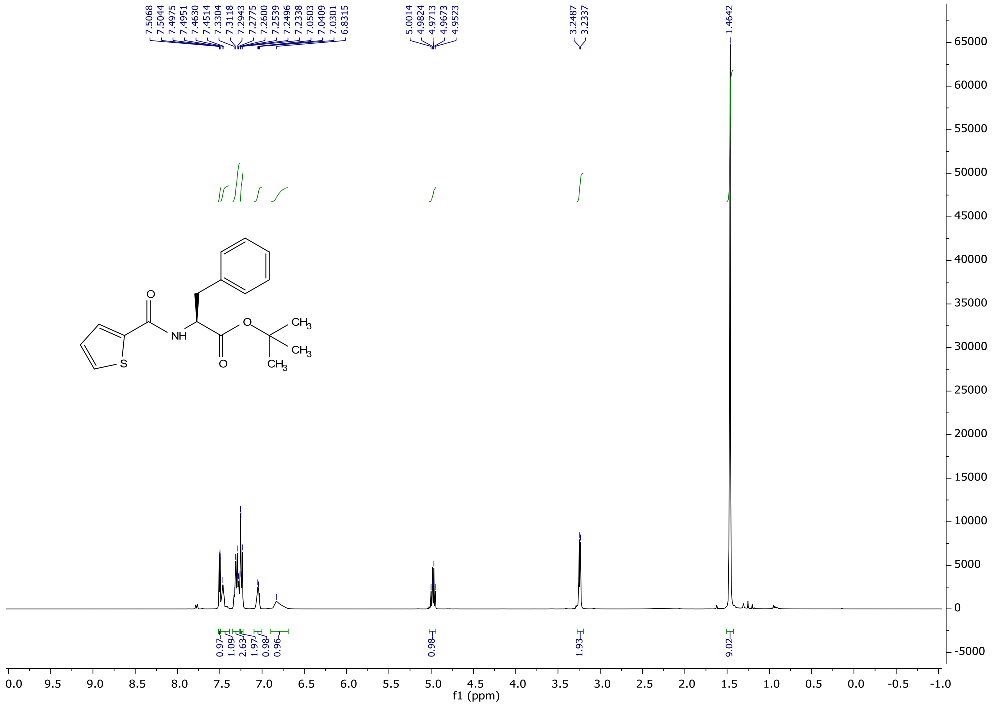

Figure S47. ^{1}H NMR (400 MHz, $CDCl_3$) spectra of **4h**.

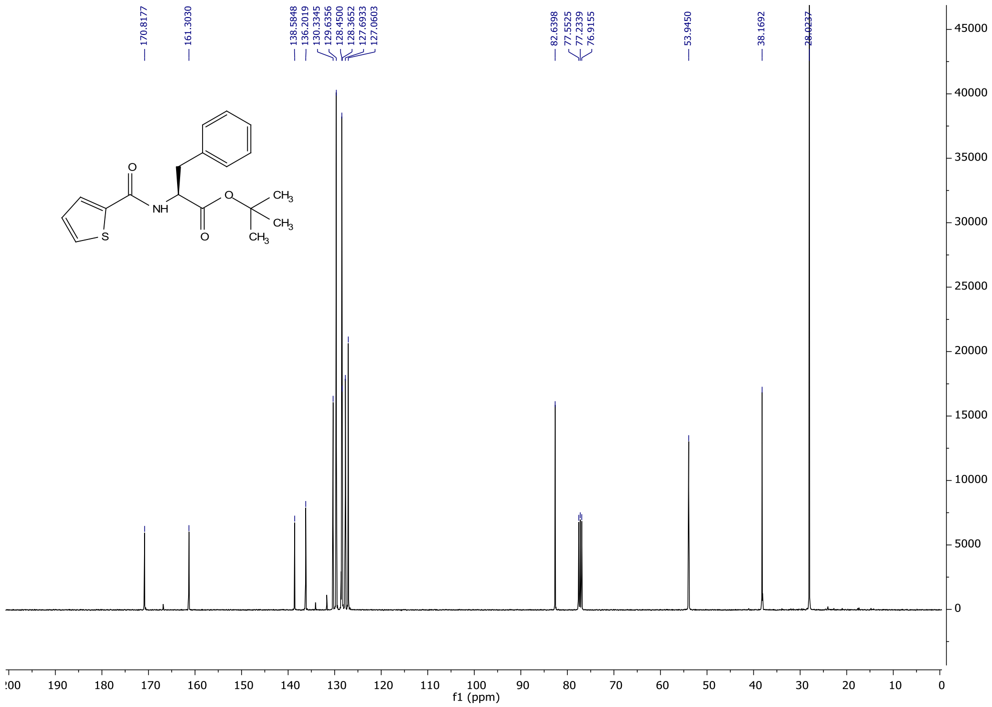

Figure S48. $^{13}C\{^1H\}$ NMR (101 MHz, $CDCl_3$) spectra of **4h**.

***Tert*-butyl (4-chlorobenzoyl)-L-phenylalaninate (4i)**

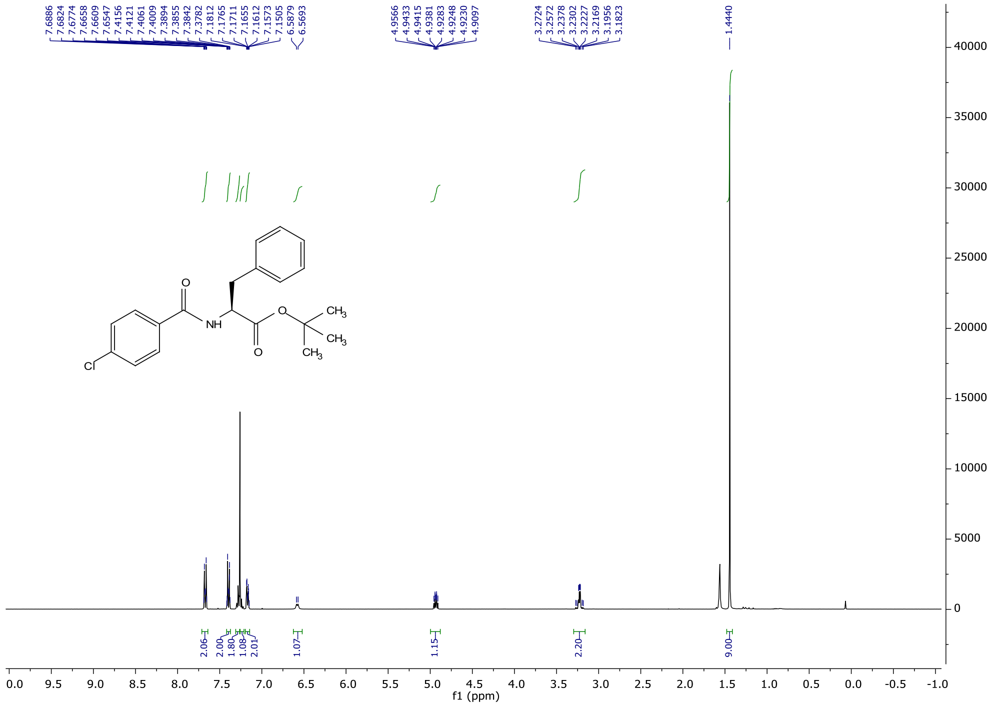

Figure S49. ^{1}H NMR (400 MHz, $CDCl_3$) spectra of **4i**.

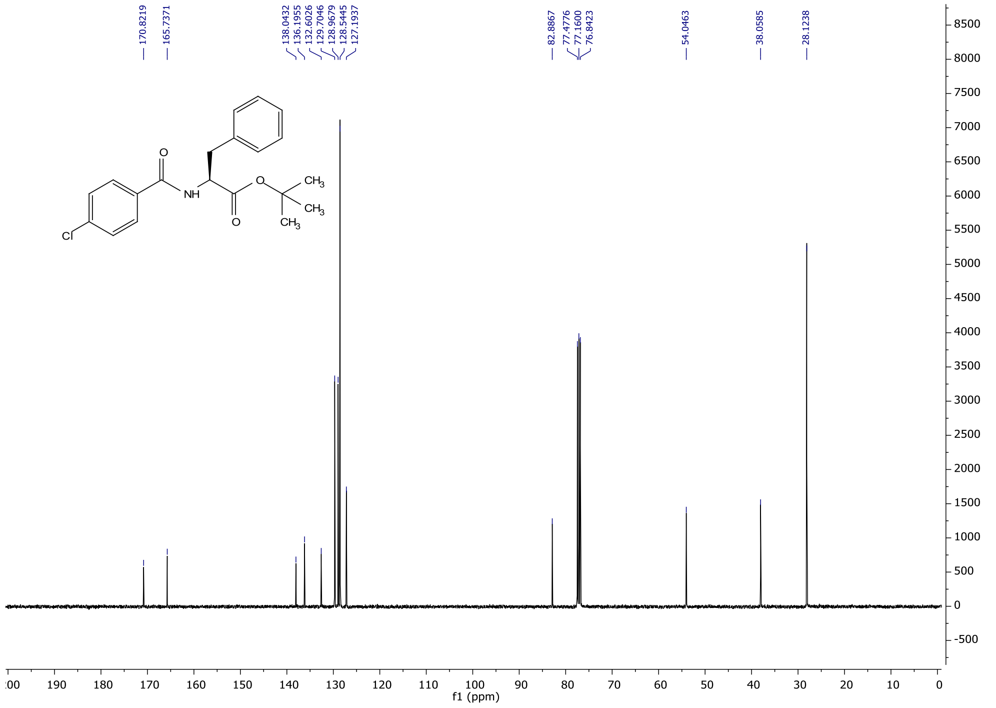

Figure S50. $^{13}C\{^1H\}$ NMR (101 MHz, $CDCl_3$) spectra of **4i**.
